# Aldosterone-Regulated Sodium Transport and Blood Pressure

**DOI:** 10.3389/fphys.2022.770375

**Published:** 2022-02-07

**Authors:** Akaki Tsilosani, Chao Gao, Wenzheng Zhang

**Affiliations:** Department of Regenerative & Cancer Cell Biology, Albany Medical College, Albany, NY, United States

**Keywords:** aldosterone, angiotensin II, ACTH, potassium, CYP11B2, ENaC, SGK1, Dot1

## Abstract

Aldosterone is a major mineralocorticoid steroid hormone secreted by glomerulosa cells in the adrenal cortex. It regulates a variety of physiological responses including those to oxidative stress, inflammation, fluid disruption, and abnormal blood pressure through its actions on various tissues including the kidney, heart, and the central nervous system. Aldosterone synthesis is primarily regulated by angiotensin II, K^+^ concentration, and adrenocorticotrophic hormone. Elevated serum aldosterone levels increase blood pressure largely by increasing Na^+^ re-absorption in the kidney through regulating transcription and activity of the epithelial sodium channel (ENaC). This review focuses on the signaling pathways involved in aldosterone synthesis and its effects on Na^+^ reabsorption through ENaC.

## Introduction

**Figure 1:**
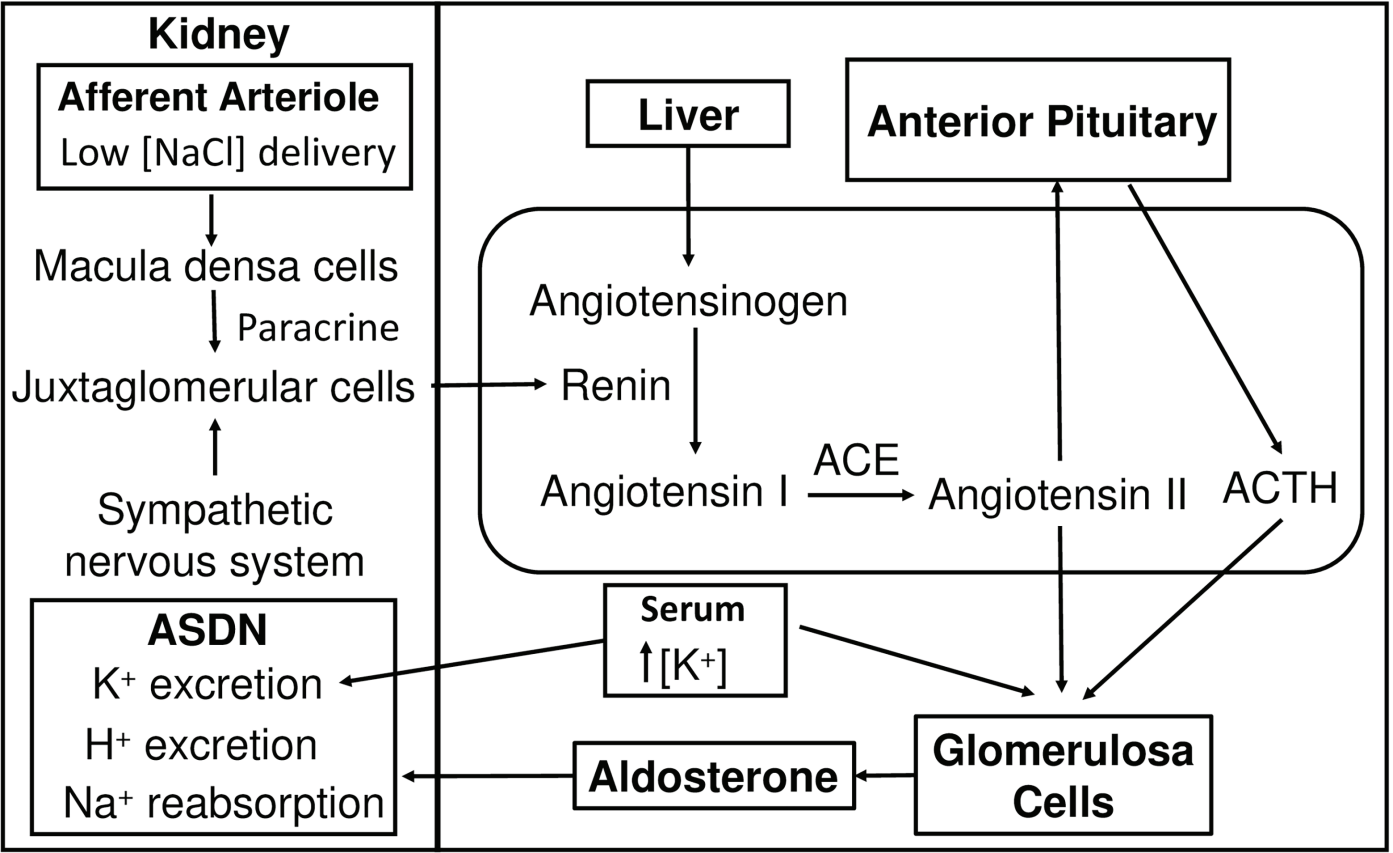
Hypotension-induced activation of the renin-angiotensin-aldosterone system. As blood pressure drops, juxtaglomerular cells receive signals from macula densa cells and the sympathetic nervous system and secrete renin into the circulation. Renin hydrolyzes liver-synthesized angiotensinogen into inactive ANG I. ANG I is converted to active ANG II by ACE. ANG II stimulates glomerulosa cells in the adrenal cortex to secrete aldosterone and the anterior pituitary gland in the brain to secrete the ACTH, which also results in aldosterone production. High K^+^ concentration stimulates aldosterone secretion from glomerulosa cells. Aldosterone increases Na^+^ reabsorption, K^+^ and H^+^ secretion in ASDN leading to an increase in blood pressure. ANG I, angiotensin I; ANG II, angiotensin II; ACE, angiotensin-converting enzyme; ASDN, aldosterone-sensitive distal nephron.

Aldosterone is a mineralocorticoid steroid hormone first isolated and characterized in 1954 which functions mainly to raise blood pressure (Simpson et al., 1954). Aldosterone has been a topic of extensive research due to its crucial role in the regulation of fluid homeostasis. The secretion of aldosterone from glomerulosa cells (GC) located in the cortex of the adrenal glands is regulated by numerous factors, but the most prominent are extracellular K^+^ concentration and the renin–angiotensin system (RAS; Bravo, 1977; Tremblay and LeHoux, 1993). Kidneys play a vital role in the initiation of RAS (Figure 1). Lowered blood pressure triggers the release of renin into the circulation from juxtaglomerular cells (JGC) in the afferent arteriole of the nephron (Friis et al., 2013). Renin release can also be triggered by the sympathetic nervous system and by decreased NaCl delivered to the distal tubule. Macula densa cells located in the juxtaglomerular apparatus sense low NaCl concentration of the filtrate and release paracrine signals that stimulate JGC (Peti-Peterdi and Harris, 2010). Renin is an aspartic protease that hydrolyzes liver-released proenzyme angiotensinogen creating angiotensin I, which undergoes further cleavage by carboxypeptidase angiotensin-converting enzyme (ACE) to create active angiotensin II (ANG II; Crisan and Carr, 2000). ANG II directly stimulates GC to secrete aldosterone. Multiple other factors are also able to regulate aldosterone synthesis, such as Klotho protein (KL), ACTH, natriuretic peptides (NPs), and circadian clock.

The nephron, the functional unit of the kidney, is the main target of aldosterone (Figure 1). Aldosterone exerts its action on the aldosterone-sensitive distal nephron (ASDN) comprising the late distal convoluted tubule (DCT2), the connecting tubule (CNT), and the collecting duct distal segments of the nephron (Bachmann et al., 1999; Reilly and Ellison, 2000). ASDN governs unidirectional Na^+^ transport from the filtrate into the circulation and bi-directional K^+^ transport (Gumz et al., 2015; Roy et al., 2015). There are two cell types in these segments: principal cells (PC) and intercalated cells (IC). PC are involved in Na^+^ and K^+^ transport while IC predominantly regulate acid–base homeostasis (Loffing and Kaissling, 2003; Roy et al., 2015). Aldosterone binds its mineralocorticoid receptor (MR; Shibata and Fujita, 2011). Almost all cells express MR, but whether they are affected by aldosterone depends on the presence of 11-β-hydroxysteroid dehydrogenase type-2 (11β-OHSD2), an enzyme that catalyzes 11-hydroxy-glucocorticoids to glucocorticoid metabolites (Funder et al., 1988). Mineralocorticoids and glucocorticoids have a common chemical structure and have equal binding affinity for MR (Arriza et al., 1987). To maintain the binding specificity in aldosterone-sensitive cells, 11β-OHSD2 catabolizes glucocorticoids rendering MR free to bind aldosterone. Both PC and IC express MR and 11β-OHSD2; however, PC has significantly higher levels of both proteins (Naray-Fejes-Toth et al., 1994; Kyossev et al., 1996). Ligand-bound MR translocates to the nucleus, where it regulates expression of its target genes (Naray-Fejes-Toth et al., 1994). Nevertheless, aldosterone also affects its target tissue through rapid non-genomic pathways (Arima et al., 2003; Funder, 2005; Funder, 2006).

Chronic elevation of aldosterone *via* intravenous injection has been demonstrated to increase arterial and mean circulatory filling pressure and resulted in significant water and sodium retention in dogs (Pan and Young, 1982). Aldosterone produces these effects by affecting electrolyte transport in both PC and IC. In PC, aldosterone regulates the expression and activity of epithelial sodium channel (ENaC) leading to increased Na^+^ reabsorption from the filtrate into the circulation. Aldosterone also has significant effects on IC. There are two main types of IC: A-type and B-type; however, non-A type and non-B type have also been described (Teng-umnuay et al., 1996). Secretion of H^+^ occurs in all types of IC through H+ ATPase and H^+^/K^+^-ATPase. H^+^/K^+^-ATPase exchanges H^+^ for K^+^ and consists of two catalytic subunits HKα_1_ and HKα_2_ (Gumz et al., 2010b). H^+^/K^+^-ATPase is located on the apical side of A-type IC and non-A and non-B IC and on basolateral side of B-type IC (Verlander et al., 1994; Roy et al., 2015). The expression of pendrin, a Na^+^ independent Cl^−^/HCO_3_^−^ exchanger is also observed in non-A and non-B IC as well as in B-type IC (Tsuruoka and Schwartz, 1999). Mineralocorticoids influence both H^+^ K^+^-ATPase and pendrin. Mineralocorticoid excess increases the expression of HKα_2_ mRNA levels, blood K^+^, and Cl^−^ and decreases blood Na^+^ and HCO_3_^+^ levels (Greenlee et al., 2011). Aldosterone upregulates pendrin expression partially through regulated IC-specific MR phosphorylation (Shibata et al., 2013a; Hirohama et al., 2018). MR has an IC-specific phosphorylation site at S843. S843 phosphorylation prevents activation of MR. ANG II stimulates MR S843 dephosphorylation to increase its binding with aldosterone (Shibata et al., 2013a).

Due to its crucial function in the regulation of blood pressure, aldosterone imbalance is implicated in many diseases. Hyperaldosteronism (Crohn’s disease) is a disease in which adrenal glands produce an excess of aldosterone leading to hypokalemia, hypertension, and chronic kidney disease (CKD; Papadopoulou-Marketou et al., 2000). In contrast, hypoaldosteronism is characterized by significantly low levels of aldosterone in the blood (DeFronzo, 1980). These two conditions represent both ends of the spectrum of diseases caused by aldosterone imbalance. Old age and obesity are part of this spectrum as they are risk factors of hypertension. Even though the principal targets of aldosterone are the epithelial cells of the kidney, it also exerts its action on non-epithelial cells of the heart, brain, and vasculature. Thus, imbalance in aldosterone levels result in cardiovascular diseases (Rocha and Funder, 2002; Yoshimoto and Hirata, 2007; Funder and Reincke, 2010; He and Anderson, 2013).

The goal of this article is to describe the recent understanding of aldosterone synthesis and its effect on electrolyte balance. Although aldosterone produces a variety of effects in multiple tissues, we focus on mechanisms by which aldosterone regulates sodium transport through ENaC in ASDN.

## Mechanisms of Aldosterone Secretion

As mentioned above, ANG II, ACTH, and K^+^ are the main signaling molecules that regulate the production of aldosterone. These inputs can have two modes of action: acute and chronic. The acute response happens within minutes and results in the rise of aldosterone due to activation of enzymes involved in the biosynthetic pathway and mobilization of cholesterol, while chronic effect takes place hours after the signal and involves alterations in gene expression.

### Aldosterone Biosynthesis Pathway

The adrenal cortex is divided into three functionally distinct regions: zona glomerulosa (production of mineralocorticoids), zona fasciculata (production of glucocorticoids), and zona reticularis (production of androgenic hormones; Vinson, 2016). Aldosterone biosynthesis occurs solely in the mitochondria of zona glomerulosa cells, which was demonstrated in the late 1980s where only isolated mitochondria of zona glomerulosa synthesized aldosterone (Ohnishi et al., 1988). This division of the adrenal cortex is crucial as adrenal steroid hormones are derived from cholesterol, thus functional zonation is one way to control the production of steroid hormones.

**Figure 2:**
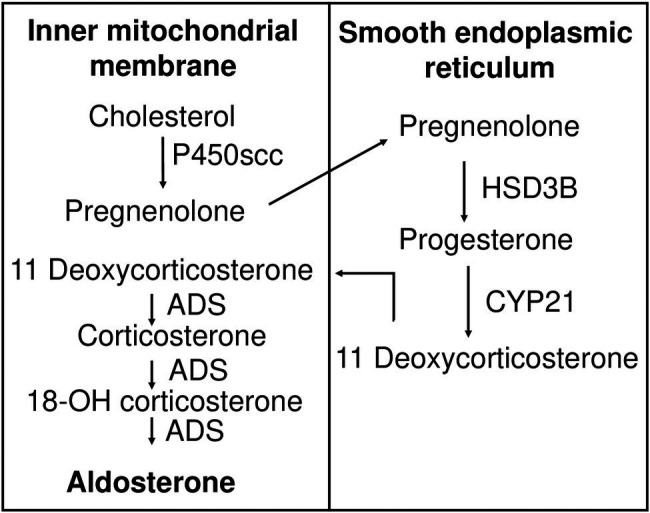
Aldosterone biosynthesis pathway. Cholesterol is transported to the inner mitochondrial membrane, where it is hydroxylated and cleaved by cytochrome P450scc to produce pregnenolone. Pregnenolone is relocated to the membrane of smooth endoplasmic reticulum, where it is oxidized by HSB3D to produce progesterone. Eleven deoxycorticosterone is generated by CYP21-mediated hydroxylation of progesterone and moves back to the inner mitochondrial membrane, where it is subject to ADS-catalyzed sequential 11-hydroxylation, 18-hydroxylation, and 18-oxidation, producing corticosterone, 18-OH corticosterone, and finally aldosterone, respectively. P450scc, cytochrome P450 side chain cleavage enzyme; HSD3B, 3β-hydroxysteroid dehydrogenase; CYP 21, 21 hydroxylase; ADS, aldosterone synthase.

Like all other steroid hormones, aldosterone is derived from cholesterol (Figure 2). The first step in aldosterone biosynthesis is the transport of cholesterol to the inner mitochondrial membrane, where the cytochrome P450scc (cholesterol side-chain cleavage enzyme, encoded by *CYP11A1*), is located (Farkash et al., 1986). Through series of hydroxylation and cleavage, P450scc converts cholesterol into pregnenolone (Hume et al., 1984). 3β-hydroxysteroid dehydrogenase (HSD3B) and 21-hydroxylase (encoded by *CYP21* gene) convert pregnenolone to 11-deoxycorticosterone (11DCS). Electron microscopy and immunohistochemistry (IHC) demonstrated that these two enzymes reside on the membrane of the smooth endoplasmic reticulum (SER; Ishimura and Fujita, 1997). The last steps of the synthesis occur in mitochondria where aldosterone synthase (ADS), encoded by the *CYP11B2* gene, accomplishes 11-hydroxylation, 18-hydroxylation, and 18-oxidation of 11DCS to produce aldosterone (Ishimura and Fujita, 1997). Thus, precursors of aldosterone are shuttled back and forth between mitochondria and SER. The actin cytoskeleton is thought to be involved in this transport (Sewer and Li, 2008). Steroid acute regulatory protein (STAR) regulates the rate-limiting step, conversion of cholesterol into pregnenolone. STAR is a 30 kDa protein that exists on the outer membrane of mitochondria and is responsible for transporting cholesterol to P450scc (Artemenko et al., 2001). STAR requires two phosphorylation events to reach its full activity (Fleury et al., 2004; Castillo et al., 2015). STAR plays a key role in aldosterone synthesis as mutations in *STAR* lead to deficiency in adrenal and gonadal aldosterone synthesis and are associated with lipoid congenital adrenal hyperplasia (Bose et al., 2000; Hasegawa et al., 2000).

Although the accepted notion is that aldosterone is produced solely by adrenal glands, some studies have shown that the heart can synthesize aldosterone in response to stress. RT-PCR analyses showed expression of *CYP11A1* and *CYP21*, the genes encoding steroidogenic enzymes involved in aldosterone synthesis, in adult human tissues (atria, ventricles, aorta apex, and intraventricular septum), and expression of *CYP11B2* in the aorta and fetal heart (Kayes-Wandover and White, 2000). Genetically hypertensive adrenalectomized and angiotensin II-treated rats had increased activity of ADS and produced aldosterone (Takeda et al., 2000). Interestingly, expression of *CYP11B2* was detectable by RT-PCR in failing human hearts, but not in normal hearts (Young et al., 2001). Bose et al. (2021) most recently reported a novel mitochondrial complex consisting of ADS, mitochondrial translocase receptor (Tom22), and STAR. This complex is responsible for the production of aldosterone in rat hearts upon stress. However, the ability of the heart to produce aldosterone is still controversial. More studies are needed to elucidate the mechanisms responsible for cardiac aldosterone synthesis.

### Angiotensin II

**Figure 3:**
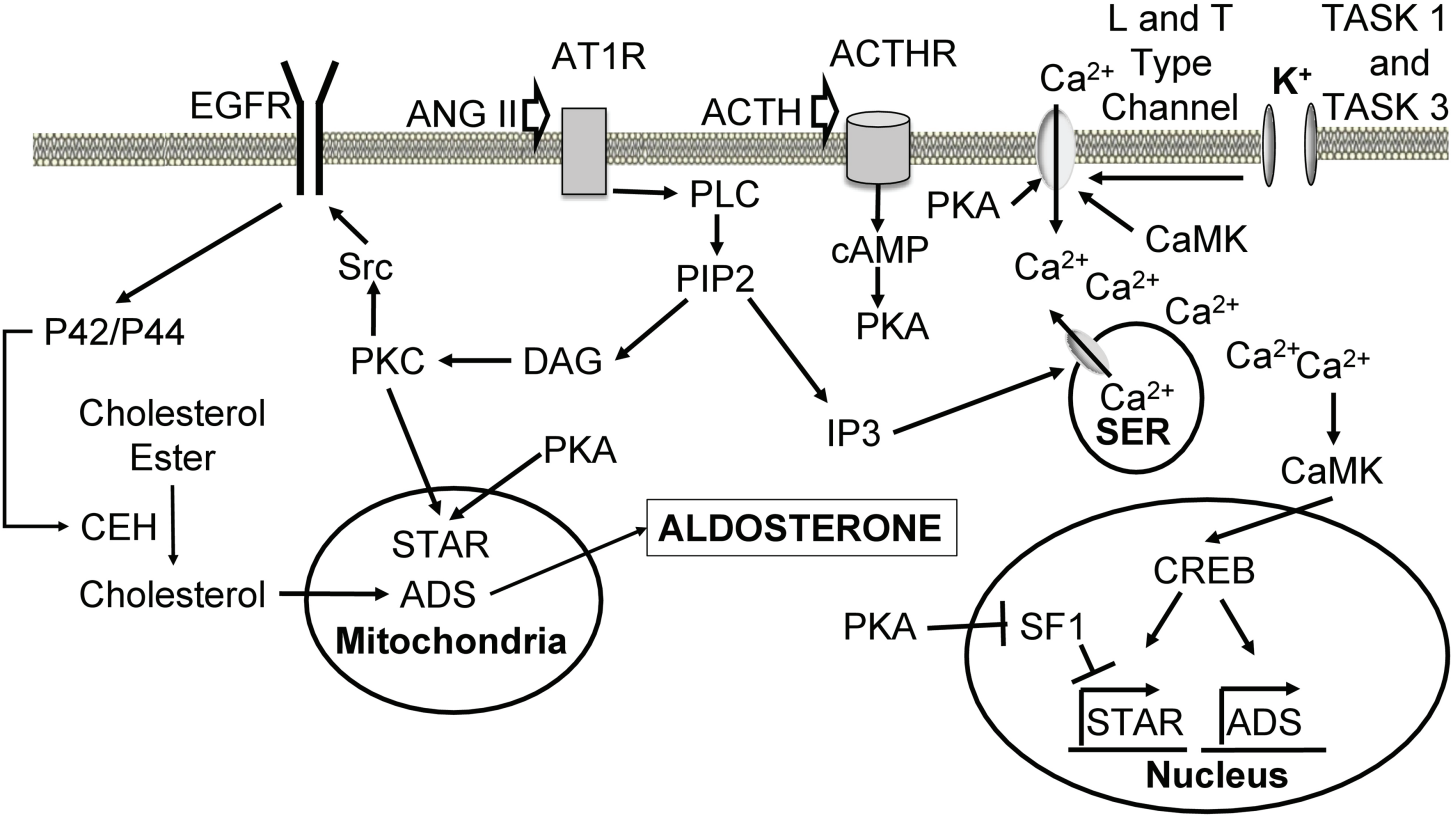
Cellular mechanisms leading to increased aldosterone production upon angiotensin II, ACTH, and K^+^ stimulation. Ang II binds to AT1R, leading to dissociation of the alpha subunit and activation of PLC. PLC hydrolyses PIP2 into DAG and IP3. IP3 binds to its receptor on the SER leading to the release of Ca^2+^ stores. Ca^2+^ activates CaMK, which causes an increase in ADS expression through CREB. DAG activates PKC to phosphorylate Src, which phosphorylates EGFR leading to activation of p42/p44 mitogen-activating protein kinase pathway. P42/p44 phosphorylates CEH to hydrolyze cholesterol esters located in the lipid droplets, making them available for transport to the inner mitochondrial membrane by STAR. PKC also phosphorylates and activates STAR. Cholesterol is used for aldosterone synthesis. ACTH binds its ACTHR leading to the activation of adenylate cyclase, which produces cAMP from ATP. cAMP triggers PKA-mediated phosphorylation and activation of STAR. PKA also phosphorylates L and T type Ca^2+^ channels causing Ca^2+^ influx. PKA increases the expression of ADS through relieving SF1-mediated inhibition of STAR. High extracellular K^+^ concentration depolarizes cells and leads to activation of L and T type Ca^2+^ channels, which allow calcium inflow from the extracellular space. ANG II, angiotensin II; AT1R, angiotensin II receptor type 1; GPCR, G protein-coupled receptor; PLC, phospholipase C; PIP2, phosphatidylinositol 4,5-bisphosphate; DAG, diacylglycerol; IP3, inositol 1,4,5 triphosphate; SER, smooth endoplasmic reticulum; CaMK, Ca^2+^/calmodulin-dependent protein kinase; ADS, aldosterone synthase; CREB, cAMP-response element binding protein; PKC, protein kinase C; EGFR, epidermal growth factor receptor; CEH, cholesterol ester hydrolase; STAR, steroid acute regulatory protein; ACTH, adrenocorticotropic hormone; ACTHR, adrenocorticotropic hormone receptor; SF1, steroidogenic factor 1.

ANG II triggers multiple signaling pathways (Figure 3) upon binding to its receptor angiotensin receptor type I (AT1), a G protein-coupled receptor (GPCR; Steckelings et al., 2010). The response of activated AT1 is similar to other GPCRs. ANG II binding leads to dissociation of GPCR subunits and activation of phospholipase C beta (PLC), which hydrolyses phosphatidylinositol-4,5-bisphosphate (PIP2) to diacylglycerol (DAG) and inositol 1,4,5-trisphosphate (IP3). IP3 interacts with IP3 receptor on SER, opening Ca^2+^ channels and resulting in a transient increase in intracellular Ca^2+^ concentration (Taylor and Thorn, 2001). ANG II also causes the influx of calcium from the extracellular space (Spat et al., 1991). Ca^2+^ is thought to increase aldosterone production through Ca^2+^/calmodulin-dependent protein kinase (CaMK).

To date, multiple CaMK have been identified (Takemoto-Kimura et al., 2017). CaMK I and II have been shown to play a role in aldosterone signaling in the adrenal gland. The role of CaMK in aldosterone production is crucial, as its inhibition abrogates the expression of ADS and aldosterone synthesis (Condon et al., 2002). IHC analysis shows that CaMK I is expressed in the adrenal cortex and transfection of adrenal cells with CaMK I coding sequence leads to increased expression of ADS (Condon et al., 2002). Compared to normal adrenal glands, aldosterone-producing adenomas (APA) have significantly higher mRNA and protein levels of CaMK I and ADS expression (Sackmann et al., 2011). KN62, a potent CaMK II inhibitor, decreased production of aldosterone in an adrenocortical tumor cell line (Clyne et al., 1995). CaMK II activation almost doubled in the presence of elevated ANG II or K^+^ levels and diminished drastically upon KN62 treatment (Fern et al., 1995). CaMK II increases Ca^2+^ entry into the cell by phosphorylating Ser1198 in the II-III loop of a α1H T-type Ca^2+^ channel (Yao et al., 2006). CaMK can be phosphorylated by CaMK kinases (CaMKK). CaMKK are also crucial regulators of ADS expression (Nanba et al., 2015). Treatment with STO-609, a specific inhibitor of CaMKK, results in decreased expression of ADS and STAR in HAC15 human adrenal cell line. To determine whether CaMKK I or II are responsible for this effect, shRNA-mediated knockdown was performed. Knockdown of CaMKK II resulted in decreased ADS expression and aldosterone production, but silencing CaMKK I had no effect. Furthermore, IHC revealed expression of CaMKK II in GC (Nanba et al., 2015). One way by which ANG II increases aldosterone synthesis is through regulating transcription of ADS. cAMP-response element binding protein (CREB), a downstream target of CaMK I and II, appears to play an important role in this process (Tokumitsu et al., 1995; Nogueira and Rainey, 2010). ANG II stimulation leads to CaMK I nuclear localization, phosphorylation of CREB, and its association with ADS promoter, while mutations of CREB diminishes the effect of ANG II on ADS mRNA levels (Bassett et al., 2000; Sackmann et al., 2011).

Diacylglycerol seems to be a key second messenger of ANG II signaling as its inhibition dampens ANG II response in normal human adrenal GC (Natarajan et al., 1988a,b, 1990). DAG appears to control aldosterone synthesis through its downstream target protein kinase C (PKC), inhibition of which reduces aldosterone production upon ANG II stimulation (Kapas et al., 1995; Wang, 2006). PKC likely promotes steroidogenesis by increasing the expression and/or activity of STAR. Phorbol 12-myristate 13-acetate (PMA) activates PKC pathway, leading to increased STAR phosphorylation and expression, and progesterone synthesis (Manna et al., 2009). Protein kinase D (PKD) also promotes STAR expression since overexpression of constitutively active PKD mutant results in upregulated STAR mRNA expression (Olala et al., 2014). Both PKC and PKD effects on STAR expression are dependent on CREB (Manna et al., 2009; Olala et al., 2014).

ANG II has also been shown to increase the local concentration of cholesterol by promoting the uptake of lipoprotein cholesterol ester, increasing local mitochondrial cholesterol concentration, and activating cholesterol ester hydrolase (CEH; Cherradi et al., 2001, 2003). PKC is considered as an important factor in these effects because PMA-activated PKC pathway mimics ANG II-induced production of aldosterone, high-density lipoprotein receptor scavenger receptor class B type I, and the low-density lipoprotein receptor in the human NCI-H295R adrenocortical cell line (Pilon et al., 2003). PKC and Ca^2+^ activate nonreceptor Src kinase resulting in transactivation of epidermal growth factor receptor (EGFR) and activation of p42/p44 mitogen-activating protein kinase (MAPK) pathway (Hodges et al., 2007). ANG II stimulation activates p42/p44 MAPK in GC (Cherradi et al., 2003). P42/p44 likely phosphorylates CEH thereby increasing the concentration of cholesterol available for aldosterone synthesis. This process may be crucial, as the phosphorylation of CEH and production of pregnenolone are reduced upon p42/p44 inhibition (Cherradi et al., 2003).

Alteration in various aspects of ANG II signaling pathways has been implicated in APA. Patients with APA and idiopathic adrenal hyperplasia (IAH) have elevated serum AT1 autoantibodies, levels of which correlate with mean arterial pressure of the patients (Rossitto et al., 2013; Li et al., 2015). High levels of aldosterone production in APA seem to be the consequence of elevated serum autoantibodies. Human adrenocortical carcinoma cells incubated with IgG isolated from APA patient’s serum-stimulated aldosterone production and *CYP11B2* expression (Piazza et al., 2019). Somatic mutations in G protein are also associated with APA. The gain of function mutation in *GNA11*, a gene coding the *α* subunit of the G protein, and its close homologue *GNAQ* have been identified in patients with APA. However, these mutations seem to be clinically silent without a codriver mutation in *CTNNB1*, a gene encoding catenin β1 (Zhou et al., 2021). The importance of aberrant activation of Wnt/β-catenin signaling pathways in APA is well characterized (Wang et al., 2017). An Increase in Ca^2+^ signaling also seems to play an important role in APA. Compared to the normal adrenal glands, APAs express higher levels of CaMKI and show increased CREB phosphorylation (Sackmann et al., 2011). Somatic mutations in *CACNA1D*, a gene encoding voltage-dependent, L type alpha 1D subunit, have been identified in APA (Azizan et al., 2013; Scholl et al., 2013). One of T-type Ca^2+^ channels, CaV3.2, is upregulated in APA and correlated with plasma aldosterone levels and *CYP11B2* expression (Felizola et al., 2014). Additionally, mutations in *CACNA1H*, a gene encoding the *α* subunit of CaV3.2 have been identified in APA and could be the cause of early-onset hypertension with primary aldosteronism (Scholl et al., 2015; Nanba et al., 2020). These mutations are thought to cause elevated Ca^2+^ influx, resulting in increased aldosterone synthesis (Reimer et al., 2016).

### K^+^

It is well known that extracellular K^+^ concentration regulates ADS expression and aldosterone synthesis (Tremblay and LeHoux, 1993). Adrenal cortex and GC express potassium channel subfamily K members 3 and 9 (KCNSK3/9, also called TASK 1/3), which play a pivotal role in this process. These “leak” channels maintain a negative resting membrane potential by producing a background K^+^ conductance (Quinn et al., 1987). However, increase in extracellular K^+^ concentration or activation of GPCR inhibits these channels causing depolarization of the membrane leading to an influx of extracellular Ca^2+^ through L and T type Ca^2+^ channels (Lymangrover et al., 1982; Kanazirska et al., 1992; Varnai et al., 1995, 1998; Horvath et al., 1998; Bandulik et al., 2010). Consistently, inhibition of calcium in GC abolishes not only the effect of potassium but also the effect of ANG II (Rossier et al., 1998; Uebele et al., 2004). Interestingly, knockout of TASK 1 disrupted the functional zonation in the adrenal cortex suggesting that K^+^ is a crucial factor in this process (Heitzmann et al., 2008). Effects of K^+^ are independent of ANG II as high K^+^ concentration was able to increase expression of ADS and the production of aldosterone in angiotensinogen knockout mice (Okubo et al., 1997). Thus, similar to ANG II, K^+^ increases aldosterone synthesis through Ca^2+^ mediated pathways described above.

Disruption in K^+^ transport in GC is implicated in multiple aldosterone-related diseases. The deletion of TASK 1 and 3 causes primary hyperaldosteronism (PH) and low-renin essential hypertension, respectively, due to constant depolarization of GC membrane in mice (Davies et al., 2008; Guagliardo et al., 2012). Mutations in another K^+^ channel, a homotetrameric inward rectifier potassium channel (KCNJ5), are associated with (APA) and PH (Ishihara et al., 2009; Choi et al., 2011; Monticone et al., 2012; Mulatero et al., 2012; Williams et al., 2015). These mutations increase aldosterone production due to altered channel selectivity leading to depolarization of the membrane (Scholl et al., 2012; Oki et al., 2012b). In fact, ANG II-mediated regulation of aldosterone synthesis can occur by downregulating the expression of KCNJ5 (Kanazirska et al., 1992). Overexpression of KCNJ5 blunts ANG II stimulatory effects on membrane potential, intracellular Ca^2+^, and expression of STAR and ADS (Oki et al., 2012a).

### Adrenocorticotropic Hormone

ACTH is released by the anterior pituitary gland and binds ACTH receptor (ACTHR), a G protein-coupled receptor, on GC. Upon ligand binding ACTHR activates adenylate cyclase and cAMP, leading to activation of protein kinase A (PKA; Fridmanis et al., 2017). ACTH induces both acute and chronic stimulatory effects on aldosterone production. *In vitro* studies show that the acute effect occurs by the action of PKA, which phosphorylates STAR and increases its expression (Jo et al., 2005). Similarly to K^+^ and ANG II, ACTH also elevates intracellular Ca^2+^ levels through PKA-mediated phosphorylation of L-type Ca^2+^ channels (Sculptoreanu et al., 1993).

The chronic response is mediated through steroidogenic factor-1 (SF1), which negatively regulates the transcription of *CYP11B2* and STAR in H295R and mouse Y1 cells (Gyles et al., 2001; Bassett et al., 2002). siRNA and shRNA-mediated silencing of SF1 drastically increased ADS expression and aldosterone production, while its overexpression elicited an opposite effect. Interestingly, these effects were observed in ANG II stimulated cells as well, suggesting that ANG II acts partially through regulating SF1 (Bassett et al., 2002; Ouyang et al., 2011). Moreover, SF1 deficient mice died shortly after birth and exhibited incomplete or absent development of adrenal glands and gonads, but showed normal expression of ADS in the placenta, which expressed both SF1 and ADS (Sadovsky et al., 1995) is phosphorylated on serine 203 by Erk1/2, resulting in its full activation (Hammer et al., 1999). The mechanism by which ACTH inhibits SF1 is not well understood. ACTH seems to have a biphasic effect on the activation of Erk1/2 and phosphorylation of SF1. Some reports show that ACTH induces Erk1/2 phosphorylation, which in turn phosphorylates SF1 abrogating its inhibitory effect on steroidogenesis (Hammer et al., 1999; Gyles et al., 2001; Le and Schimmer, 2001; Winnay and Hammer, 2006). On the other hand, ACTH-induced PKA activation led to *de novo* synthesis and activation of mitogen-activated protein kinase phosphatase 1 (MKP1), which dephosphorylated both SF1 and Erk1/2 (Bey et al., 2003; Sewer and Waterman, 2003; Winnay and Hammer, 2006). Both of these pathways seem to be important for aldosterone synthesis, as silencing of either Erk1/2 or MKP1 reduces steroidogenesis (Gyles et al., 2001; Sewer and Waterman, 2003).

While it is clear that ACTH induces aldosterone synthesis, this effect seems to be transient. At first ACTH increases aldosterone synthesis of GC cells; however, after continuous induction by ACTH, GC phenotype changes to that of zona fasciculata leading to a decrease in aldosterone synthesis (Crivello and Gill, 1983). *In vivo* findings are consistent with these results. Since ACTH is released in a pulsatile fashion in humans, Seely et al. (1989) investigated the effect of pulsatile and prolonged infusion of ACTH on aldosterone levels (Seely et al., 1989). Pulsatile infusion resulted in an increase and maintenance of aldosterone, while prolonged infusion led to sharp increase followed by a continuous decrease in aldosterone levels (Seely et al., 1989). These effects cannot be explained by sodium, potassium, angiotensin-II, or cortisol as their levels were the same in both groups, thus the mechanisms that govern these effects remain unknown. GC ADS mRNA levels were significantly increased and then dramatically decreased at 3 and 24 h after ACTH treatment in rats, respectively (Holland and Carr, 1993). Chronic infusion of ACTH for 2–3 weeks resulted in disappearance of GC and consequently a decrease in aldosterone production (Mitani et al., 1996). Similar transient effects of ACTH on aldosterone levels are seen in human male subjects (Fuchs-Hammoser et al., 1980).

Plasma renin and aldosterone follow a circadian rhythm because their levels fluctuated throughout the day, with their levels being highest in the mornings and lowest in the evenings in normal men (Cugini et al., 1981; Thosar et al., 2019). Similar results have been found in PA and essentially hypertensive patients (Kem et al., 1973; Lamarre-Cliche et al., 2005). Interestingly, plasma aldosterone circadian rhythm (PACR) may be under androgenic rather than renin control, as aldosterone acrophase precedes renin and is associated with cortisol (Stern et al., 1986). Early studies in rats and human males found that dexamethasone treatment, a drug that suppresses ACTH, abolishes normal PACR, suggesting that it is under ACTH control (Hilfenhaus, 1976; Takeda et al., 1984). Multiple regression analysis of aldosterone-stimulating factors at 3 hourly intervals confirmed PACR dependence on ACTH rather than renin or ANG II (Takeda et al., 1984). The role of ACTH in PACR has also been implicated in PA (Sonoyama et al., 2014).

### Klotho, Leptin, Natriuretic Peptides, and Circadian Rhythm

Although ANG II, K^+^, and ACTH are thought to be the main stimulators of aldosterone production, there are other factors that can regulate this process, some of which are prominent in hypertensive conditions. Klotho protein (KL) is a single-pass transmembrane type 1 glycoprotein which has been regarded as an anti-aging molecule because as age increases serum KL levels decrease (Zhou et al., 2016). Low serum KL levels are associated with age-related disorders, such as coronary artery disease, atherosclerosis, myocardial infarction, and hypertension (Olejnik et al., 2018). Fischer et al. (2010) demonstrated the negative correlation between serum KL and aldosterone levels in mice. Hypomorphic KL (KL^+/−^) mice showed increased ACTH, antidiuretic hormone (ADH), and aldosterone levels compared to WT. Interestingly, Ca^2+^ deficient diet alleviated the symptoms of hyperaldosteronism in KL^+/−^ (Fischer et al., 2010). Overexpression of KL reduces aldosterone production while impaired expression of KL increases aldosterone production (Zhou et al., 2016). KL half deficiency seems to produce these effects by increasing the expression of ADS (Zhou et al., 2016). Similarly, there was a positive correlation between serum aldosterone level and CKD stage and a negative correlation between serum KL and aldosterone levels in human CKD patients, suggesting that the effect of KL on aldosterone in humans is similar to mice (Qian et al., 2018). Nevertheless, it remains unclear how a decrease in KL abundance results in an increase in aldosterone synthesis. One possibility is that KL acts as a negative regulator of aldosterone biosynthesis. This hypothesis can be tested *in vitro*. Reduced expression of key genes involved in aldosterone synthesis (such as ADS and STAR) as well as aldosterone levels in GC lines treated with KL would support this hypothesis.

Obesity is a well-known cause of hypertension and is characterized by high aldosterone levels (Goodfriend et al., 1998; Kurukulasuriya et al., 2011). One possibility is that adipocytes affect aldosterone production since they are active endocrine tissues (Ronti et al., 2006). Indeed, Ehrhart-Bornstein et al. (2003) showed that isolated adipocyte secretory products could dramatically increase aldosterone production independent of ANG II in adrenocortical cells (NCI-H295R; Ehrhart-Bornstein et al., 2003). 2,13-epoxy-9-keto-10 (trans)-octadecenoic acid (EKODE) has also been shown to increase aldosterone production in a GC line. EKODE is produced by the oxidation of linoleic acid by hepatocytes. Incubation of adrenal cells with EKODE increased aldosterone production independently of ANG II. Interestingly, adult humans have a positive correlation with blood EKODE and aldosterone levels (Goodfriend et al., 2004). However, EKODE is unlikely the molecule responsible for the effect seen by Ehrhart-Bornstein et al. (2003), as adipocyte secretory products were not oxidized by hepatocytes. A subsequent study showed that adipocyte-derived factors from SHR/cp rats (model of metabolic syndrome with hypertension) stimulate aldosterone production by increasing ADS expression and STAR activation despite ANG II receptor inhibition. Adipocyte-derived factors from normal rats failed to replicate these results (Nagase et al., 2006). These effects might be mediated by leptin, which is a protein hormone secreted by adipocytes and is abnormally high in obese individuals (Martinez-Rumayor et al., 2008; Huby et al., 2015). These *in vitro* studies have been validated and extended by *in vivo* investigations. For example, leptin infusion increased expression of ADS and serum aldosterone in a dose-dependent manner in mice with no effect on ANG II, K^+^, and corticosterone levels (Belin de Chantemele et al., 2011; Huby et al., 2015). Huby et al. (2015) concluded that “leptin is a new regulatory factor of aldosterone secretion that acts directly in the adrenal cortex to promote ADS expression and aldosterone production” (Huby et al., 2015). The leptin stimulatory effect on ADS and aldosterone was not abolished upon administration of ANG II or β adrenergic receptor inhibitors in mice, further supporting the notion of leptin as a novel effector of aldosterone production (Huby et al., 2015). Leptin achieves these effects possibly through CaMK II, as leptin increased intracellular Ca^2+^ concentration and elevated expression calmodulin and CaMK II (Huby et al., 2015). Agreeably administration of leptin receptor antagonism abrogated leptin-mediated aldosterone secretion and lowered blood pressure in mice (Huby et al., 2016). These studies carry crucial importance as hypertension in the obese population is a devastating health issue (Kurukulasuriya et al., 2011).

Natriuretic peptides (NPs), cardiovascular peptides mostly secreted by the heart, play a role in vasodilation and fluid homeostasis. NPs have autocrine and paracrine signaling abilities and can function as endocrine components (Martinez-Rumayor et al., 2008). Due to their role in blood pressure, they have been hypothesized to regulate aldosterone secretion. Indeed, peptides in heart’s crude extracts were able to inhibit aldosterone production by GC even upon ANG II and ACTH stimulation (Atarashi et al., 1984). Consequent studies confirmed these results *in vivo* and showed that atrial NPs dampen aldosterone response to ANG II in rats (Chartier et al., 1984; Atarashi et al., 1985). Similar effects were seen in human males. Administration of ANG II or ACTH alone raised blood pressure and plasma aldosterone levels. Simultaneous infusion of low levels of atrial NPs along with either ACTH or ANG II produced no significant change in blood pressure or aldosterone levels (Anderson et al., 1986; Weidmann et al., 1986; Cuneo et al., 1987). Another way by which NPs regulate blood pressure is by affecting renal filtration and renin release. Isolated rabbit afferent arterioles and suspended JGC exposed to NPs showed drastic decrease in renin secretion (Itoh et al., 1987; Takagi et al., 1988). *In vivo* studies in dogs are consistent with these results as atrial NP infusion increased renal flow, glomerular filtration rate, sodium and potassium excretion and reduced blood pressure and renin production (Burnett et al., 1984; Maack et al., 1984). These results suggest that RAS and NPs may act as endogenous antagonists.

Circadian clock controls many physiological functions, such as blood pressure, immune response, and metabolism, potentially through four “circadian clock” proteins: period 1–3 (Per 1–3), Bmal1, Clock cryptochrome 1–2, and Clock (Eckel-Mahan and Sassone-Corsi, 2009; Agarwal, 2010; Bollinger et al., 2010; Dibner et al., 2010). Per1 regulates expression of αENaC in both aldosterone-dependent and-independent manners (Gumz et al., 2009, 2010a; Richards et al., 2013). It also coordinately regulates the expression of other genes involved in renal Na^+^ reabsorption. These include Per1-mediated upregulation of Na^+^-K^+^-ATPase through Fxyd5 and downregulation of endothelin 1, which is a potential inhibitor of ENaC (Lubarski et al., 2005; Bugaj et al., 2008). Per-1 not only controls downstream targets of aldosterone, but also the plasma levels of aldosterone itself. This is supported by the findings in Per-1 knockout mice. Ablation of Per1 in mice led to decreased aldosterone and 3β-dehydrogenase isomerase levels (Richards et al., 2013). Interestingly, male mice appear to be more susceptible to adverse phenotypes of Per-1 KO than female mice. Treatment of Per-1 KO mice maintained on high salt diet with desoxycorticosterone pivalate (DOCP), an aldosterone analog, lead to increased mean arterial pressure and loss of normal circadian blood pressure (Solocinski et al., 2017). These effects are not observed in female Per-1 KO mice with similar treatments (Douma et al., 2019). This difference can be explained by endothelin 1. Male mice under high salt diet and DOCP treatment had decreased night/day ratio of urinary ET-1 and different ET-1 and ET-1 receptor gene expression compared to female mice (Douma et al., 2020).

## Mechanisms of Aldosterone Action

Upon binding to aldosterone, MR undergoes conformational changes, leading to dissociation from chaperone proteins, dimerization, and translocation to the nucleus, where it binds to the responsive elements in the promoter regions of target genes to regulate transcription. These changes in gene expression play a major role in the regulation of blood pressure, which is accomplished through the control of sodium reabsorption by regulating either transcription or the activity of the ENaC.

### Epithelial Sodium Channel

Epithelial sodium channel is a highly selective Na^+^ channel that is expressed on the apical membrane of various epithelial tissues, such as ASDN, colon, lungs, and sweat glands. ENaC is specific to Na^+^ over other ions, such as K^+^ and highly sensitive to diuretic amiloride. In the kidney, ENaC is exclusively expressed by principal cells where it reabsorbs Na^+^ from the filtrate. Na^+^ is then transported into the bloodstream by Na^+^/K^+^ ATPase located on the basolateral side leading to an increase in extracellular fluid volume and subsequently an increase in blood pressure (Pan and Young, 1982; Garty and Palmer, 1997).

Epithelial sodium channel is comprised of three subunits: *α*, *β*, and *ϒ* (Canessa et al., 1994). Although all three subunits are required for full functionality, the stoichiometric ratio of the subunits is still unclear. Originally it was thought that ENaC forms a tetramer with 2α, 1β, and 1ϒ subunits (Firsov et al., 1998; Dijkink et al., 2002; Anantharam and Palmer, 2007), but recent evidence suggests a 1:1:1 stoichiometric ratio (Staruschenko et al., 2005; Kashlan and Kleyman, 2011; Noreng et al., 2018). Each subunit spans the PM twice with both the COOH and NH_2_ termini oriented toward the cytoplasm (Noreng et al., 2018). The COOH terminus of each subunit contains a PY domain that plays a crucial role in ENaC regulation. Deletions or mutations of this domain causes Liddle syndrome, a hereditary disease characterized by abnormally high ENaC activity and expression to the PM leading to hypertension (Firsov et al., 1996; Staub et al., 1996). For example, truncation or frameshift mutations in the COOH terminus of the βENaC were identified in subjects with Liddle syndrome (Shimkets et al., 1994) In contrast, mutations of the conserved glycine residues in the NH_2_ terminus result in pseudohypoaldosteronism type 1 (PHA I), a life-threatening disease characterized by salt wasting, hyperkalemia, and metabolic acidosis (Chang et al., 1996).

Since ENaC dysfunction can be fatal, ENaC activity is tightly regulated. ENaC is primarily regulated by controlling its presence in the PM. ENaC is delivered to the PM through clathrin-mediated exocytosis and is removed from the PM through ubiquitylation. However, Na^+^ transport is also regulated through proteolytic cleavage of ENaC (Rossier and Stutts, 2009). Multiple proteases have been shown to increase activity of ENaC including serine, cysteine, furin, and alkaline proteases (Chraibi et al., 1998; Hughey et al., 2004; Butterworth et al., 2012; Haerteis et al., 2012). Increase in activity of ENaC by proteolytic cleavage is achieved by releasing a 43-amino acid inhibitory domain of γ-subunit (Zachar et al., 2015). For a more comprehensive review please refer to (Kleyman and Eaton, 2020).

### Serum Glucocorticoid-Induced Kinase 1

One of the keyways by which aldosterone regulates ENaC is through a serine/threonine serum glucocorticoid-induced kinase 1 (SGK1). SGK1 expression was increased 60 min post-injection of physiological dose of aldosterone (Chen et al., 1999; Bhargava et al., 2001). Although the levels of SGK1 rise in the presence of aldosterone, it must be phosphorylated at Thr256 and Ser422 by pyruvate dehydrogenase kinase 1 (PDK1) to be fully active (Park et al., 1999). Phosphorylation of a third highly conserved residue (Ser397) also increased SGK1 activity (Chen et al., 2009). mTORC2 was also identified as a kinase for SGK1 and is required for ENaC activation (Lu et al., 2010).

**Figure 4:**
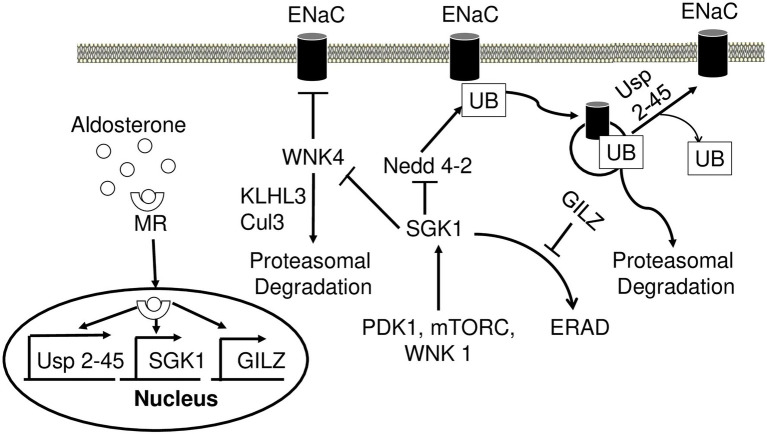
Aldosterone regulates epithelial sodium channel (ENaC) activity and degradation. Aldosterone-bound MR translocates to the nucleus and induces transcription of USP 2-45, SGK1, and GILZ. SGK1 phosphorylates WNK4 and dampens its inhibitory action on ENaC activity. Nedd4-2 ubiquitinates ENaC and signals it for proteasomal degradation. Wnk4 is targeted to proteasomal degradation by KLHL3-Cul3 ubiquitin ligase. SGK1 inhibits this process by phosphorylating Nedd4-2 reducing its affinity to ENaC. USP2-45 removes UB from ENaC preventing its degradation. SGK1 requires phosphorylation events in order to achieve full activity, which is accomplished by PDK1, Wnk1, and mTORC. In the absence of aldosterone, SGK1 is subject to ERAD. However, in the presence of aldosterone GILZ inhibits this process increasing the stability of SGK1. MR, mineralocorticoid receptor; SGK1, serum glucocorticoid-induced kinase 1; GILZ, glucocorticoid-induced leucine zipper 1; Nedd4-2, Neural precursor cell expressed developmentally downregulated gene 4; ENaC, epithelial sodium channel; UB, ubiquitin; PDK1, pyruvate dehydrogenase kinase; ERAD, endoplasmic reticulum-associated degradation.

Neural precursor cell expressed developmentally downregulated gene 4 (Nedd4-2) is a ubiquitin ligase that plays a crucial role in regulating ENaC (Figure 4). As mentioned above, the main mechanism by which the cell regulates Na^+^ transport is by controlling the number of channels in the PM. In the absence of aldosterone, Nedd4-2 decreases this number by ubiquitinylating the PY motif of all three ENaC subunits and signaling the complex for degradation (Zhou et al., 2007). This mechanism is thought to be responsible for the development of Liddle syndrome, as the mutations of the PY motif prevent ubiquitination and lead to an increased number of ENaC in the PM (Rotin, 2008). In the presence of aldosterone, SGK1 phosphorylates Nedd4-2, impairing Nedd4-2 binding to ENaC, and instead increasing its affinity for 14–3–3 (Debonneville et al., 2001; Bhalla et al., 2005). Alongside SGK1, a deubiquitylating enzyme Usp2-45 also seems to be an important regulator of ENaC. Usp2-45 is upregulated upon aldosterone induction and de-ubiquitinates ENaC leading to higher cell surface expression of the channel (Fakitsas et al., 2007; Verrey et al., 2008).

WNK4 is a serine/threonine kinase, mutations of which have been identified as a potential cause for PHA II (Wilson et al., 2001; Lopez-Cayuqueo et al., 2018). The underlying mechanism behind this disease may be explained by a negative regulation of ENaC through WNK4 (Figure 4). Both *in vivo* and *in vitro* studies have shown a significant reduction of ENaC surface expression upon interacting with WNK4 (Ring et al., 2007a). ENaC-WNK4 interaction requires an intact COOH terminus of β and ϒ subunits but not the PY motif, differing from ENaC-Nedd4-2 interaction requiring the PY motif. In the presence of aldosterone, SGK1 phosphorylates WNK4 and abrogates its negative regulation of ENaC (Ring et al., 2007a,b; Yu et al., 2013). The clinical relevance of ENaC-WNK4 interaction is illustrated by PHA II-associated R1185C mutation of WNK4, which decreases WNK4’s inhibitory effect on ENaC by enhancing SGK1-mediated phosphorylation of WNK4 at S1217 (Na et al., 2013). Aldosterone also increases the expression of kidney-specific WNK1 (kinase-deficient variant), which consequently increases transepithelial Na^+^ transport in cortical collecting duct cells potentially through regulation of ENaC (Naray-Fejes-Toth et al., 2004). WNK1 appears to increase ENaC surface expression by activating SGK1 through a non-catalytic mechanism (Xu et al., 2005a,b). This appears to be dependent on phosphatidylinositol 3-kinase, as its inhibition abrogates this effect (Xu et al., 2005b). Both WNK4 and WNK1 are implicated in PHA II (Wilson et al., 2001). Two other genes, *KLHL3* and *CUL3*, encoding kelch-like 3 (Kelch) and cullin 3 (cul3) proteins, respectively, may explain the mechanism by which WNK4 and WNK1 cause PHA II. Cul3 is an integral member of cul3-RING ubiquitin ligase, an E3 ubiquitin ligase. It forms a scaffold for the RING finger protein and ubiquitin conjugating enzyme E2 (Genschik et al., 2013). Kelch is an adaptor protein that connects cul3-RING ubiquitin ligase to its targets (Ji and Prive, 2013). Mutations in *KLHL3* and *CUL3* have been implicated in PHA II and appear to cause hypertension and electrolyte disbalance (Boyden et al., 2012; Louis-Dit-Picard et al., 2012). One mechanism by which these mutations cause PHA II is through Wnk1 and Wnk4, as both of these proteins are targets of Cul3-RING ubiquitin ligase (Ohta et al., 2013; Shibata et al., 2013b). PHA II causing mutations in KLHL3 decreases Wnk4 binding to Cul3-RING ubiquitin ligase, decreasing WNK4 degradation and increasing its levels resulting in hypertension (Mori et al., 2013; Wakabayashi et al., 2013; Wu and Peng, 2013; Susa et al., 2014).

SGK1 is expressed in many tissues, but it has a short half-life under basal conditions (Brickley et al., 2002). Upon aldosterone stimulation, A6 cells dramatically increased SGK1 expression (Chen et al., 1999). SGK1 contains a short hydrophobic motif that targets the protein to the ER where it is degraded by ER-associated degradation (ERAD). The deletion of this motif redistributes the protein into the cytoplasm and increases its half-life (Brickley et al., 2002; Arteaga et al., 2006; Belova et al., 2006; Bogusz et al., 2006). In the presence of aldosterone, this negative regulation of SGK1 is abrogated due to the action of glucocorticoid-induced leucine zipper 1 (GILZ1), the levels of which rise in the presence of the steroid hormone (Soundararajan et al., 2005). GILZ1 reduces ER localization of SGK1 and recruits it to ENaC leading to significantly lower ERAD of SGK1 and higher levels of Na^+^ transport (Soundararajan et al., 2010; Rashmi et al., 2017).

### Dot1a

In addition to controls of proteolytic cleavage and subcellular localization, ENaC is also regulated at the transcriptional level *via* the disruptor of telomeric silencing 1 (Dot1), a histone H3 K79 methyltransferase. Dot1 can mono, di or tri- methylate H3 K79 leading to a wide range of epigenetic control of gene expression. Dot1 is implicated in complex cellular processes, such as cell cycle regulation, cell proliferation, DNA replication, apoptosis, telomeric silencing, and blood pressure control. Dot1 has at least five isoforms (a–e) created by alternative splicing, out of which Dot1a is the most prominent in mouse kidneys (Nguyen and Zhang, 2011). Deletion of *Dot1* specifically in the connecting tubules and collecting ducts facilitated development of kidney fibrosis and reduced kidney function under three experimental settings (streptozotocin-induced diabetes, during normal aging, and after unilateral ureteral obstruction) in mice (Zhang et al., 2020).

**Figure 5:**
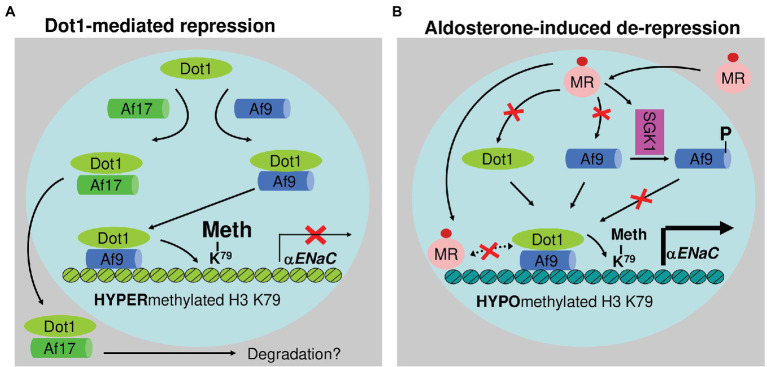
Epigenetic control of αENaC transcription. Under basal conditions **(A)**, Af9 recruits Dot1a to form a nuclear complex, which indirectly or directly through Af9 DNA-binding activity binds specific sites of the αENaC promoter, leading to hypermethylation of histone H3 K79 and repression of αENaC transcription. Af17 relieves the repression by competing with Af9 for binding Dot1a and promoting Dot1a redistribution from the nucleus to cytoplasm. In the presence of aldosterone **(B)**, αENaC transcription is induced by a variety of mechanisms. Through the classical action, aldosterone binds and activates the mineralocorticoid receptor to bind the glucocorticoid response element in the αENaC promoter and transactivate αENaC. In parallel, aldosterone releases Dot1a–Af9-mediated repression by reducing the formation of the complex through three mechanisms: downregulating Dot1a and Af9 expression presumably via nuclear receptor-dependent or -independent (not shown) mechanisms, decreasing the Dot1a–Af9 interaction via SGK1-mediated phosphorylation of Af9 at Ser435, and counterbalancing Dot1a–Af9 complex by activating MR to compete for binding Af9. These actions collectively result in histone H3 K79 hypomethylation at specific subregions of the αENaC promoter. In all cases, Af9-free Dot1a binds DNA nonspecifically and catalyzes histone H3 K79 methylation throughout the genome under basal conditions (not shown). Revised from Chen et al. (2015). Dot1a: disruptor of telomeric silencing 1a. Meth: methylation. αENaC: α epithelial sodium channel. NR: nuclear hormone receptor. SGK1: serum glucocorticoid-induced kinase 1.

Dot1a interacts with Af9, a putative transcription factor. Under basal conditions, Dot1a-Af9 complex binds to the specific regions of *αENaC*, where it promotes H3 K79 methylation associated with promoter and represses *αENaC* transcription (Zhang et al., 2007). Aldosterone downregulates Dot1a and Af9 expression and impairs Dot1a-Af9 interaction by SGK1-mediated Af9 phosphorylation. Consequently, the abundance of Dot1a-Af9 complex at the *αENaC* is reduced, leading to histone H3 K79 hypomethylation and derepression of *αENaC* (Zhang et al., 2007). Dot1a-Af9 complex is also negatively regulated under the basal condition through Af17, another Dot1a binding partner. Af17 competes with Af9 for binding Dot1a and facilitates Dot1a nuclear export into the cytoplasm for possible degradation, resulting in relief of Dot1a-Af9-mediated repression and an increase in *αENaC* expression (Figure 5; Reisenauer et al., 2009; Wu et al., 2011). Analyses of *Af17^−/−^* mice illustrated the significance of Dot1a-Af9-Af17 complexes in Na^+^ and blood pressure handing (Chen et al., 2011). *Af17^−/−^* vs. WT mice had elevated histone H3 K79 methylation at the *αENaC* promoter and reduced ENaC function. The impaired ENaC function stemmed from reduced ENaC expression at both mRNA and protein levels, fewer active channels, lower open probability, and decreased effective activity. As a result, *Af17^−/−^* vs. WT mice displayed lower blood pressure, higher urine volume, and more sodium excretion in spite of mildly increased plasma concentrations of aldosterone. Af17 deficiency with respect to sodium handling and blood pressure, however, was completely compensated by high levels of plasma aldosterone induced by multiple methods (Chen et al., 2011). Hence, Af17 is considered as a potential locus for the maintenance of sodium and BP homeostasis and H3K79 methylation is directly linked to these processes. The potential genetic–epigenetic interplay of DOT1-AF9-AF17 in human blood pressure control was well-reviewed (Zhang et al., 2013).

## Discussion

Aldosterone is a vital steroid hormone produced by the adrenal glands that regulates blood pressure by affecting electrolyte and fluid balance. Aldosterone is synthesized from cholesterol in the mitochondria and SER of GC upon decreased blood pressure, although some reports suggest that heart tissue is also capable of aldosterone secretion. Lowered blood pressure results in activation of ANG II. ANG II binds its receptor on GC, resulting in the production of IP3 and DAG. IP3 and DAG raise intracellular Ca^2+^ concentration and activate PKC and p42/p44 MAPK pathway, respectively. Ca^2+^ activates CaMK, which stimulates the expression of ADS. PKC and p42/p44 are involved in the activation of STAR and CEH increasing the rate of aldosterone production. High extracellular K^+^ concentration also stimulates aldosterone synthesis. At physiological serum K^+^ levels, K^+^ moves out of the GC through TASK 1 and three maintaining negative membrane potential. However, in the presence of ANG II or high extracellular K^+^ concentration, TASK 1 and 3 are inhibited which causes depolarization of the cell leading to the entry of extracellular Ca^2+^. These initiate similar signaling pathways as ANG II leading to aldosterone synthesis. ACTH has both acute and chronic effects on aldosterone synthesis. It activates PKA, which phosphorylates STAR and activates it. ACTH transiently stimulates aldosterone production by increasing intracellular Ca^2+^ levels. ACTH is thought to mediate ADS expression by affecting the activity of SF1; however, the mechanism is not fully understood. Aldosterone secretion is regulated by other, nontraditional factors, such as KL, leptin, natriuretic peptides, and circadian clock. KL is a protein that is associated with aging as its levels decrease with age and inversely correlates with age-related disorders. Studies in mice show that deficiency in KL stimulates aldosterone production by the adrenal gland in an ANG II-independent manner. Clinical studies in CKD patients confirm the negative correlation between KL and ANG II serum levels. Leptin is a hormone produced by adipocytes and its plasma leptin levels are high in obese patients. Leptin binds to the leptin receptor on GC and stimulates the secretion of aldosterone by activating CaMK pathway. Hypertension in obese individuals is often independent of ANG II, K^+^, and ACTH concentrations. Since leptin increases aldosterone production despite inhibition of ANG II and ACTH receptors, it can explain the phenomenon seen in obese individuals. Natriuretic peptides are thought to be endogenous antagonists to RAS as their administration decreases aldosterone production despite ANG II or ACTH stimulation. Aldosterone levels tend to rise in the morning and fall in the evening, suggesting the role of PACR. One of core circadian clock proteins, Per-1 has been shown to regulate not only Na^+^ transport in ASDN, but also plasma aldosterone levels.

Aldosterone stimulates Na^+^ transport by regulating the expression and activity of ENaC. Aldosterone stimulates the expression and stability of SGK1, which directly and indirectly increases the expression and activity of ENaC. SGK1 phosphorylates Nedd4-2, a ubiquitin ligase that ubiquitinates a PY motif of ENaC and targets it for degradation. Upon phosphorylation by SGK1, Nedd4-2 loses its affinity to ENaC thereby increasing the number of channels in the PM. SGK1 also phosphorylates WNK4, a negative regulator of ENaC activity. Upon phosphorylation by SGK1, WNK4 weakens its interaction with ENaC. SGK1 itself is expressed in many tissues but is immediately targeted for degradation by ERAD. Aldosterone prevents its degradation by increasing the expression of GILZ, which reduces ER localization of SGK1 and directs it to ENaC. Dot1a-Af9-Af17-mediated epigenetic control of ENaC and Na^+^ handling is regulated in aldosterone-dependent and -independent manners. The former involves reduction of Dot1a-Af9 complex formation through aldosterone-induced downregulation of Dot1a and Af9 and SGK1-mediated Af9 phosphorylation. The latter is achieved by competitive protein–protein interactions between Dot1a-Af9 and Dot1a-Af17.

## Author Contributions

AT and WZ: writing. CG, AT, and WZ: review and editing. WZ: supervision. All authors contributed to the article and approved the submitted version.

## Funding

This work was supported by the following grants: National Institutes of Health Grants DK080236 (to WZ).

## Conflict of Interest

The authors declare that the research was conducted in the absence of any commercial or financial relationships that could be construed as a potential conflict of interest.

## Publisher’s Note

All claims expressed in this article are solely those of the authors and do not necessarily represent those of their affiliated organizations, or those of the publisher, the editors and the reviewers. Any product that may be evaluated in this article, or claim that may be made by its manufacturer, is not guaranteed or endorsed by the publisher.

## Abbreviations

ACE: Angiotensin-converting enzyme
ACTH: Adrenocorticotropic hormone
ACTHR: Adrenocorticotrophic hormone receptor
ADH: Antidiuretic hormone
ADS: Aldosterone synthase
ANG II: Angiotensin II; APA, Aldosterone-producing adenomas
ASDN: Aldosterone-sensitive distal nephron
AT1: Angiotensin receptor type I
CaMK: Ca^2+^/Calmodulin-dependent protein kinase
CEH: Cholesterol ester hydrolase
CKD: Chronic kidney disease
CREB: cAMP-response binding element-binding protein
Cul3: Cullin 3
CYP21-21: Hydroxylase
DAG: Diacylglycerol
DOCP: Desoxycorticosterone pivalate
Dot1: Disruptor of telomeric silencing 1
EGFR: Epidermal growth factor receptor
EKODE: 2,13-epoxy-9-keto-10(trans)-octadecenoic acid
ENaC: Epithelial sodium channel
ERAD: Endoplasmic reticulum-associated degradation
ET-1: Endothelin 1
GC: Glomerulosa cell
GPCR: G protein-coupled receptor
HSB3D-3β: Hydroxysteroid dehydrogenase
ICL: Intercalated cells
IHC: Immunohistochemistry
IP3: Inositol 1,4,5 triphosphate
JGC: Juxtaglomerular cells
KCNJ5: Inward rectifier potassium channel
KCNSK3/9: Potassium channel subfamily K members 3 and 9
KL: Klotho protein
Kelch3: Kelch-like 3
MAPK: Mitogen activating protein kinase
MR: Mineralocorticoid receptor
Nedd4-2: Neural precursor cell expressed developmentally downregulated gene 4
PC: Principal cells
PDK1: Pyruvate dehydrogenase kinase 1
PH: Primary hyperaldosteronism
PHA: Pseudohypoaldosteronism type 1
PIP2: Phosphatidylinositol 4,5-bisphosphate
PKA: Protein kinase A
PKC: Protein kinase C
PKD: Protein kinase D
PM: Plasma membrane
PMA: Phorbol 12-myrstate 13-acetate
PLC: Phospholipase C
P450scc: Cholesterol side-chain cleavage enzyme
RAS: Renin angiotensin system
RT-PCR: Reverse transcription-polymerase chain reaction
SER: Smooth endoplasmic reticulum
SF1: Steroidogenic factor 1
StaR: Steroid acute regulatory protein
Tom22: Mitochondrial translocase receptor
WNK: With no lysine kinase
11DCS-11: Deoxycorticosterone
11β-OHSD2-11: β-hydroxysteroid dehydrogenase type-2

## References

[ref1] Agarwal R. (2010). Regulation of circadian blood pressure: from mice to astronauts. Curr. Opin. Nephrol. Hypertens. 19, 51–58. doi: 10.1097/MNH.0b013e3283336ddb, PMID: 19864947 PMC2876349

[ref2] Anantharam A. Palmer L. G. (2007). Determination of epithelial Na^+^ channel subunit stoichiometry from single-channel conductances. J. Gen. Physiol. 130, 55–70. doi: 10.1085/jgp.200609716, PMID: 17562820 PMC2154365

[ref3] Anderson J. V. Struthers A. D. Payne N. N. Slater J. D. Bloom S. R. (1986). Atrial natriuretic peptide inhibits the aldosterone response to angiotensin II in man. Clin. Sci. 70, 507–512. 10.1042/cs0700507 2938872

[ref4] Arima S. Kohagura K. Xu H. L. Sugawara A. Abe T. Satoh F.. (2003). Nongenomic vascular action of aldosterone in the glomerular microcirculation. J. Am. Soc. Nephrol. 14, 2255–2263. doi: 10.1097/01.ASN.0000083982.74108.54, PMID: 12937301

[ref5] Arriza J. L. Weinberger C. Cerelli G. Glaser T. M. Handelin B. L. Housman D. E.. (1987). Cloning of human mineralocorticoid receptor complementary DNA: structural and functional kinship with the glucocorticoid receptor. Science 237, 268–275. 3037703 10.1126/science.3037703

[ref6] Arteaga M. F. Wang L. Ravid T. Hochstrasser M. Canessa C. M. (2006). An amphipathic helix targets serum and glucocorticoid-induced kinase 1 to the endoplasmic reticulum-associated ubiquitin-conjugation machinery. Proc. Natl. Acad. Sci. U. S. A. 103, 11178–11183. doi: 10.1073/pnas.0604816103, PMID: 16847254 PMC1544061

[ref7] Artemenko I. P. Zhao D. Hales D. B. Hales K. H. Jefcoate C. R. (2001). Mitochondrial processing of newly synthesized steroidogenic acute regulatory protein (StAR), but not total StAR, mediates cholesterol transfer to cytochrome P450 side chain cleavage enzyme in adrenal cells. J. Biol. Chem. 276, 46583–46596. doi: 10.1074/jbc.M107815200 11579102

[ref8] Atarashi K. Mulrow P. J. Franco-Saenz R. (1985). Effect of atrial peptides on aldosterone production. J. Clin. Invest. 76, 1807–1811. 2997288 10.1172/JCI112172 PMC424212

[ref9] Atarashi K. Mulrow P. J. Franco-Saenz R. Snajdar R. Rapp J. (1984). Inhibition of aldosterone production by an atrial extract. Science 224, 992–994. 6326267 10.1126/science.6326267

[ref10] Azizan E. A. Poulsen H. Tuluc P. Zhou J. Clausen M. V. Lieb A.. (2013). Somatic mutations in ATP1A1 and CACNA1D underlie a common subtype of adrenal hypertension. Nat. Genet. 45, 1055–1060. doi: 10.1038/ng.2716, PMID: 23913004

[ref11] Bachmann S. Bostanjoglo M. Schmitt R. Ellison D. H. (1999). Sodium transport-related proteins in the mammalian distal nephron – distribution, ontogeny and functional aspects. Anat. Embryol. 200, 447–468. 10.1007/s004290050294 10526014

[ref12] Bandulik S. Penton D. Barhanin J. Warth R. (2010). TASK1 and TASK3 potassium channels: determinants of aldosterone secretion and adrenocortical zonation. Horm. Metab. Res. 42, 450–457. doi: 10.1055/s-0029-1243601, PMID: 20049674

[ref13] Bassett M. H. Zhang Y. Clyne C. White P. C. Rainey W. E. (2002). Differential regulation of aldosterone synthase and 11beta-hydroxylase transcription by steroidogenic factor-1. J. Mol. Endocrinol. 28, 125–135. doi: 10.1677/jme.0.0280125, PMID: 11932209

[ref14] Bassett M. H. Zhang Y. White P. C. Rainey W. E. (2000). Regulation of human CYP11B2 and CYP11B1: comparing the role of the common CRE/Ad1 element. Endocr. Res. 26, 941–951. doi: 10.3109/07435800009048620, PMID: 11196473

[ref15] Belin de Chantemele E. J. Mintz J. D. Rainey W. E. Stepp D. W. (2011). Impact of leptin-mediated sympatho-activation on cardiovascular function in obese mice. Hypertension 58, 271–279. doi: 10.1161/HYPERTENSIONAHA.110.168427, PMID: 21690486 PMC3141100

[ref16] Belova L. Sharma S. Brickley D. R. Nicolarsen J. R. Patterson C. Conzen S. D. (2006). Ubiquitin-proteasome degradation of serum- and glucocorticoid-regulated kinase-1 (SGK-1) is mediated by the chaperone-dependent E3 ligase CHIP. Biochem. J. 400, 235–244. doi: 10.1042/BJ20060905, PMID: 16895519 PMC1652829

[ref17] Bey P. Gorostizaga A. B. Maloberti P. M. Lozano R. C. Poderoso C. Cornejo Maciel F.. (2003). Adrenocorticotropin induces mitogen-activated protein kinase phosphatase 1 in Y1 mouse adrenocortical tumor cells. Endocrinology 144, 1399–1406. doi: 10.1210/en.2002-220987, PMID: 12639923

[ref18] Bhalla V. Daidie D. Li H. Pao A. C. LaGrange L. P. Wang J.. (2005). Serum- and glucocorticoid-regulated kinase 1 regulates ubiquitin ligase neural precursor cell-expressed, developmentally down-regulated protein 4-2 by inducing interaction with 14-3-3. Mol. Endocrinol. 19, 3073–3084. doi: 10.1210/me.2005-0193, PMID: 16099816

[ref19] Bhargava A. Fullerton M. J. Myles K. Purdy T. M. Fundero J. W. Pearce D.. (2001). The serum- and glucocorticoid-induced kinase is a physiological mediator of aldosterone action. Endocrinology 142, 1587–1594. doi: 10.1210/endo.142.4.8095, PMID: 11250940

[ref20] Bogusz A. M. Brickley D. R. Pew T. Conzen S. D. (2006). A novel N-terminal hydrophobic motif mediates constitutive degradation of serum- and glucocorticoid-induced kinase-1 by the ubiquitin-proteasome pathway. FEBS J. 273, 2913–2928. doi: 10.1111/j.1742-4658.2006.05304.x, PMID: 16817852

[ref21] Bollinger T. Bollinger A. Oster H. Solbach W. (2010). Sleep, immunity, and circadian clocks: a mechanistic model. Gerontology 56, 574–580. doi: 10.1159/000281827, PMID: 20130392

[ref22] Bose H. S. Sato S. Aisenberg J. Shalev S. A. Matsuo N. Miller W. L. (2000). Mutations in the steroidogenic acute regulatory protein (StAR) in six patients with congenital lipoid adrenal hyperplasia. J. Clin. Endocrinol. Metab. 85, 3636–3639. doi: 10.1210/jc.85.10.3636, PMID: 11061515

[ref23] Bose H. S. Whittal R. M. Marshall B. Rajapaksha M. Wang N. P. Bose M.. (2021). A novel mitochondrial complex of aldosterone synthase, steroidogenic acute regulatory protein, and Tom22 synthesizes aldosterone in the rat heart. J. Pharmacol. Exp. Ther. 377, 108–120. doi: 10.1124/jpet.120.000365, PMID: 33526603

[ref24] Boyden L. M. Choi M. Choate K. A. Nelson-Williams C. J. Farhi A. Toka H. R.. (2012). Mutations in kelch-like 3 and cullin 3 cause hypertension and electrolyte abnormalities. Nature 482, 98–102. doi: 10.1038/nature10814, PMID: 22266938 PMC3278668

[ref25] Bravo E. L. (1977). Regulation of aldosterone secretion: current concepts and newer aspects. Adv. Nephrol. Necker Hosp. 7, 105–120. 208405

[ref26] Brickley D. R. Mikosz C. A. Hagan C. R. Conzen S. D. (2002). Ubiquitin modification of serum and glucocorticoid-induced protein kinase-1 (SGK-1). J. Biol. Chem. 277, 43064–43070. doi: 10.1074/jbc.M207604200, PMID: 12218062

[ref27] Bugaj V. Pochynyuk O. Mironova E. Vandewalle A. Medina J. L. Stockand J. D. (2008). Regulation of the epithelial Na^+^ channel by endothelin-1 in rat collecting duct. Am. J. Physiol. Renal Physiol. 295, F1063–F1070. doi: 10.1152/ajprenal.90321.2008, PMID: 18667482 PMC2576140

[ref28] Burnett J. C. Jr. Granger J. P. Opgenorth T. J. (1984). Effects of synthetic atrial natriuretic factor on renal function and renin release. Am. J. Phys. 247, F863–F866. 10.1152/ajprenal.1984.247.5.F863 6238539

[ref29] Butterworth M. B. Zhang L. Heidrich E. M. Myerburg M. M. Thibodeau P. H. (2012). Activation of the epithelial sodium channel (ENaC) by the alkaline protease from *Pseudomonas aeruginosa*. J. Biol. Chem. 287, 32556–32565. doi: 10.1074/jbc.M112.369520, PMID: 22859302 PMC3463336

[ref30] Canessa C. M. Schild L. Buell G. Thorens B. Gautschi I. Horisberger J. D.. (1994). Amiloride-sensitive epithelial Na+ channel is made of three homologous subunits. Nature 367, 463–467. 8107805 10.1038/367463a0

[ref31] Castillo A. F. Orlando U. Helfenberger K. E. Poderoso C. Podesta E. J. (2015). The role of mitochondrial fusion and StAR phosphorylation in the regulation of StAR activity and steroidogenesis. Mol. Cell. Endocrinol. 408, 73–79. doi: 10.1016/j.mce.2014.12.011, PMID: 25540920

[ref32] Chang S. S. Grunder S. Hanukoglu A. Rosler A. Mathew P. M. Hanukoglu I.. (1996). Mutations in subunits of the epithelial sodium channel cause salt wasting with hyperkalaemic acidosis, pseudohypoaldosteronism type 1. Nat. Genet. 12, 248–253. 8589714 10.1038/ng0396-248

[ref33] Chartier L. Schiffrin E. Thibault G. Garcia R. (1984). Atrial natriuretic factor inhibits the stimulation of aldosterone secretion by angiotensin II, ACTH and potassium in vitro and angiotensin II-induced steroidogenesis in vivo. Endocrinology 115, 2026–2028. 6092045 10.1210/endo-115-5-2026

[ref34] Chen S. Y. Bhargava A. Mastroberardino L. Meijer O. C. Wang J. Buse P.. (1999). Epithelial sodium channel regulated by aldosterone-induced protein sgk. Proc. Natl. Acad. Sci. U. S. A. 96, 2514–2519. 10051674 10.1073/pnas.96.5.2514 PMC26816

[ref35] Chen W. Chen Y. Xu B. E. Juang Y. C. Stippec S. Zhao Y.. (2009). Regulation of a third conserved phosphorylation site in SGK1. J. Biol. Chem. 284, 3453–3460. doi: 10.1074/jbc.M807502200, PMID: 19068477 PMC2635031

[ref36] Chen L. Wu H. Pochynyuk O. M. Reisenauer M. R. Zhang Z. Huang L.. (2011). Af17 deficiency increases sodium excretion and decreases blood pressure. J. Am. Soc. Nephrol. 22, 1076–1086. doi: 10.1681/ASN.2010121270, PMID: 21546577 PMC3103727

[ref37] Chen L. Zhang X. Zhang W. (2015). Regulation of alphaENaC transcription. Vitam. Horm. 98, 101–135. doi: 10.1016/bs.vh.2014.12.004, PMID: 25817867 PMC4867546

[ref38] Cherradi N. Bideau M. Arnaudeau S. Demaurex N. James R. W. Azhar S.. (2001). Angiotensin II promotes selective uptake of high density lipoprotein cholesterol esters in bovine adrenal glomerulosa and human adrenocortical carcinoma cells through induction of scavenger receptor class B type I. Endocrinology 142, 4540–4549. doi: 10.1210/endo.142.10.8412, PMID: 11564720

[ref39] Cherradi N. Pardo B. Greenberg A. S. Kraemer F. B. Capponi A. M. (2003). Angiotensin II activates cholesterol ester hydrolase in bovine adrenal glomerulosa cells through phosphorylation mediated by p42/p44 mitogen-activated protein kinase. Endocrinology 144, 4905–4915. doi: 10.1210/en.2003-0325, PMID: 12960096

[ref40] Choi M. Scholl U. I. Yue P. Bjorklund P. Zhao B. Nelson-Williams C.. (2011). K+ channel mutations in adrenal aldosterone-producing adenomas and hereditary hypertension. Science 331, 768–772. doi: 10.1126/science.1198785, PMID: 21311022 PMC3371087

[ref41] Chraibi A. Vallet V. Firsov D. Hess S. K. Horisberger J. D. (1998). Protease modulation of the activity of the epithelial sodium channel expressed in *Xenopus oocytes*. J. Gen. Physiol. 111, 127–138. 9417140 10.1085/jgp.111.1.127 PMC1887769

[ref42] Clyne C. D. Nguyen A. Rainey W. E. (1995). The effects of KN62, a Ca^2+^/calmodulin-dependent protein kinase II inhibitor, on adrenocortical cell aldosterone production. Endocr. Res. 21, 259–265. doi: 10.3109/07435809509030441, PMID: 7588388

[ref43] Condon J. C. Pezzi V. Drummond B. M. Yin S. Rainey W. E. (2002). Calmodulin-dependent kinase I regulates adrenal cell expression of aldosterone synthase. Endocrinology 143, 3651–3657. doi: 10.1210/en.2001-211359, PMID: 12193581

[ref44] Crisan D. Carr J. (2000). Angiotensin I-converting enzyme: genotype and disease associations. J. Mol. Diagn. 2, 105–115. doi: 10.1016/S1525-1578(10)60624-1, PMID: 11229513 PMC1906907

[ref45] Crivello J. F. Gill G. N. (1983). Induction of cultured bovine adrenocortical zona glomerulosa cell 17-hydroxylase activity by ACTH. Mol. Cell. Endocrinol. 30, 97–107. 6301909 10.1016/0303-7207(83)90204-6

[ref46] Cugini P. Scavo D. Cornelissen G. Lee J. Y. Meucci T. Halberg F. (1981). Circadian rhythms of plasma renin, aldosterone and cortisol on habitual and low dietary sodium intake. Horm. Res. 15, 7–27. 7037589 10.1159/000179430

[ref47] Cuneo R. C. Espiner E. A. Nicholls M. G. Yandle T. G. Livesey J. H. (1987). Effect of physiological levels of atrial natriuretic peptide on hormone secretion: inhibition of angiotensin-induced aldosterone secretion and renin release in normal man. J. Clin. Endocrinol. Metab. 65, 765–772. 2821056 10.1210/jcem-65-4-765

[ref48] Davies L. A. Hu C. Guagliardo N. A. Sen N. Chen X. Talley E. M.. (2008). TASK channel deletion in mice causes primary hyperaldosteronism. Proc. Natl. Acad. Sci. U. S. A. 105, 2203–2208. doi: 10.1073/pnas.0712000105, PMID: 18250325 PMC2538899

[ref49] Debonneville C. Flores S. Y. Kamynina E. Plant P. J. Tauxe C. Thomas M. A.. (2001). Phosphorylation of Nedd4-2 by Sgk1 regulates epithelial Na(+) channel cell surface expression. EMBO J. 20, 7052–7059. doi: 10.1093/emboj/20.24.7052, PMID: 11742982 PMC125341

[ref50] DeFronzo R. A. (1980). Hyperkalemia and hyporeninemic hypoaldosteronism. Kidney Int. 17, 118–134. 6990088 10.1038/ki.1980.14

[ref51] Dibner C. Schibler U. Albrecht U. (2010). The mammalian circadian timing system: organization and coordination of central and peripheral clocks. Annu. Rev. Physiol. 72, 517–549. doi: 10.1146/annurev-physiol-021909-135821, PMID: 20148687

[ref52] Dijkink L. Hartog A. van Os C. H. Bindels R. J. (2002). The epithelial sodium channel (ENaC) is intracellularly located as a tetramer. Pflugers Arch. 444, 549–555. doi: 10.1007/s00424-002-0855-4, PMID: 12136275

[ref53] Douma L. G. Crislip G. R. Cheng K. Y. Barral D. Masten S. Holzworth M.. (2020). Knockout of the circadian clock protein PER1 results in sex-dependent alterations of ET-1 production in mice in response to a high-salt diet plus mineralocorticoid treatment. Can. J. Physiol. Pharmacol. 98, 579–586. doi: 10.1139/cjpp-2019-0688, PMID: 32437627 PMC7605171

[ref54] Douma L. G. Solocinski K. Holzworth M. R. Crislip G. R. Masten S. H. Miller A. H.. (2019). Female C57BL/6J mice lacking the circadian clock protein PER1 are protected from nondipping hypertension. Am. J. Physiol. Regul. Integr. Comp. Physiol. 316, R50–R58. doi: 10.1152/ajpregu.00381.2017, PMID: 30427705 PMC6383492

[ref55] Eckel-Mahan K. Sassone-Corsi P. (2009). Metabolism control by the circadian clock and vice versa. Nat. Struct. Mol. Biol. 16, 462–467. doi: 10.1038/nsmb.1595, PMID: 19421159 PMC4073609

[ref56] Ehrhart-Bornstein M. Lamounier-Zepter V. Schraven A. Langenbach J. Willenberg H. S. Barthel A.. (2003). Human adipocytes secrete mineralocorticoid-releasing factors. Proc. Natl. Acad. Sci. U. S. A. 100, 14211–14216. doi: 10.1073/pnas.2336140100, PMID: 14614137 PMC283571

[ref57] Fakitsas P. Adam G. Daidie D. van Bemmelen M. X. Fouladkou F. Patrignani A.. (2007). Early aldosterone-induced gene product regulates the epithelial sodium channel by deubiquitylation. J. Am. Soc. Nephrol. 18, 1084–1092. doi: 10.1681/ASN.2006080902, PMID: 17344426

[ref58] Farkash Y. Timberg R. Orly J. (1986). Preparation of antiserum to rat cytochrome P-450 cholesterol side chain cleavage, and its use for ultrastructural localization of the immunoreactive enzyme by protein A-gold technique. Endocrinology 118, 1353–1365. 3948785 10.1210/endo-118-4-1353

[ref59] Felizola S. J. Maekawa T. Nakamura Y. Satoh F. Ono Y. Kikuchi K.. (2014). Voltage-gated calcium channels in the human adrenal and primary aldosteronism. J. Steroid Biochem. Mol. Biol. 144, 410–416. doi: 10.1016/j.jsbmb.2014.08.012, PMID: 25151951

[ref60] Fern R. J. Hahm M. S. Lu H. K. Liu L. P. Gorelick F. S. Barrett P. Q. (1995). Ca^2+^/calmodulin-dependent protein kinase II activation and regulation of adrenal glomerulosa Ca^2+^ signaling. Am. J. Phys. 269, F751–F760. 10.1152/ajprenal.1995.269.6.F751 8594869

[ref61] Firsov D. Gautschi I. Merillat A. M. Rossier B. C. Schild L. (1998). The heterotetrameric architecture of the epithelial sodium channel (ENaC). EMBO J. 17, 344–352. 9430626 10.1093/emboj/17.2.344 PMC1170385

[ref62] Firsov D. Schild L. Gautschi I. Merillat A. M. Schneeberger E. Rossier B. C. (1996). Cell surface expression of the epithelial Na channel and a mutant causing Liddle syndrome: a quantitative approach. Proc. Natl. Acad. Sci. U. S. A. 93, 15370–15375. 8986818 10.1073/pnas.93.26.15370 PMC26411

[ref63] Fischer S. S. Kempe D. S. Leibrock C. B. Rexhepaj R. Siraskar B. Boini K. M.. (2010). Hyperaldosteronism in klotho-deficient mice. Am. J. Physiol. Renal Physiol. 299, F1171–F1177. doi: 10.1152/ajprenal.00233.2010, PMID: 20719979 PMC3774497

[ref64] Fleury A. Mathieu A. P. Ducharme L. Hales D. B. LeHoux J. G. (2004). Phosphorylation and function of the hamster adrenal steroidogenic acute regulatory protein (StAR). J. Steroid Biochem. Mol. Biol. 91, 259–271. doi: 10.1016/j.jsbmb.2004.04.010, PMID: 15336703

[ref65] Fridmanis D. Roga A. Klovins J. (2017). ACTH receptor (MC2R) specificity: what do we know about underlying molecular mechanisms? Front. Endocrinol. 8:13. doi: 10.3389/fendo.2017.00013 PMC5292628 28220105

[ref66] Friis U. G. Madsen K. Stubbe J. Hansen P. B. Svenningsen P. Bie P.. (2013). Regulation of renin secretion by renal juxtaglomerular cells. Pflugers Arch. 465, 25–37. doi: 10.1007/s00424-012-1126-7 22733355

[ref67] Fuchs-Hammoser R. Schweiger M. Oelkers W. (1980). The effect of chronic low-dose infusion of ACTH (1-24) on renin, renin-substrate, aldosterone and other corticosteroids in sodium replete and deplete man. Acta Endocrinol. 95, 198–206. 10.1530/acta.0.0950198 6254307

[ref68] Funder J. W. (2005). The nongenomic actions of aldosterone. Endocr. Rev. 26, 313–321. doi: 10.1210/er.2005-0004 15814845

[ref69] Funder J. W. (2006). Minireview: aldosterone and the cardiovascular system: genomic and nongenomic effects. Endocrinology 147, 5564–5567. doi: 10.1210/en.2006-0826 16946012

[ref70] Funder J. W. Pearce P. T. Smith R. Smith A. I. (1988). Mineralocorticoid action: target tissue specificity is enzyme, not receptor, mediated. Science 242, 583–585. 2845584 10.1126/science.2845584

[ref71] Funder J. W. Reincke M. (2010). Aldosterone: a cardiovascular risk factor? Biochim. Biophys. Acta 1802, 1188–1192. doi: 10.1016/j.bbadis.2010.08.005 20713154

[ref72] Garty H. Palmer L. G. (1997). Epithelial sodium channels: function, structure, and regulation. Physiol. Rev. 77, 359–396. 9114818 10.1152/physrev.1997.77.2.359

[ref73] Genschik P. Sumara I. Lechner E. (2013). The emerging family of CULLIN3-RING ubiquitin ligases (CRL3s): cellular functions and disease implications. EMBO J. 32, 2307–2320. doi: 10.1038/emboj.2013.173, PMID: 23912815 PMC3770339

[ref74] Goodfriend T. L. Ball D. L. Egan B. M. Campbell W. B. Nithipatikom K. (2004). Epoxy-keto derivative of linoleic acid stimulates aldosterone secretion. Hypertension 43, 358–363. doi: 10.1161/01.HYP.0000113294.06704.64, PMID: 14718355

[ref75] Goodfriend T. L. Egan B. M. Kelley D. E. (1998). Aldosterone in obesity. Endocr. Res. 24, 789–796. doi: 10.3109/07435809809032689 9888579

[ref76] Greenlee M. M. Lynch I. J. Gumz M. L. Cain B. D. Wingo C. S. (2011). Mineralocorticoids stimulate the activity and expression of renal H^+^, K^+^_–_ATPases. J. Am. Soc. Nephrol. 22, 49–58. doi: 10.1681/ASN.2010030311, PMID: 21164026 PMC3014034

[ref77] Guagliardo N. A. Yao J. Hu C. Schertz E. M. Tyson D. A. Carey R. M.. (2012). TASK-3 channel deletion in mice recapitulates low-renin essential hypertension. Hypertension 59, 999–1005. doi: 10.1161/HYPERTENSIONAHA.111.189662, PMID: 22493079 PMC3357084

[ref78] Gumz M. L. Cheng K. Y. Lynch I. J. Stow L. R. Greenlee M. M. Cain B. D.. (2010a). Regulation of alphaENaC expression by the circadian clock protein period 1 in mpkCCD(c14) cells. Biochim. Biophys. Acta 1799, 622–629. doi: 10.1016/j.bbagrm.2010.09.003 20868778 PMC2975761

[ref79] Gumz M. L. Lynch I. J. Greenlee M. M. Cain B. D. Wingo C. S. (2010b). The renal H+-K+-ATPases: physiology, regulation, and structure. Am. J. Physiol. Renal Physiol. 298, F12–F21. doi: 10.1152/ajprenal.90723.2008, PMID: 19640897 PMC2806118

[ref80] Gumz M. L. Rabinowitz L. Wingo C. S. (2015). An integrated view of potassium homeostasis. N. Engl. J. Med. 373, 1787–1788. doi: 10.1056/NEJMc1509656, PMID: 26510039

[ref81] Gumz M. L. Stow L. R. Lynch I. J. Greenlee M. M. Rudin A. Cain B. D.. (2009). The circadian clock protein period 1 regulates expression of the renal epithelial sodium channel in mice. J. Clin. Invest. 119, 2423–2434. doi: 10.1172/JCI36908, PMID: 19587447 PMC2719945

[ref82] Gyles S. L. Burns C. J. Whitehouse B. J. Sugden D. Marsh P. J. Persaud S. J.. (2001). ERKs regulate cyclic AMP-induced steroid synthesis through transcription of the steroidogenic acute regulatory (StAR) gene. J. Biol. Chem. 276, 34888–34895. doi: 10.1074/jbc.M102063200, PMID: 11410589

[ref83] Haerteis S. Krappitz M. Bertog M. Krappitz A. Baraznenok V. Henderson I.. (2012). Proteolytic activation of the epithelial sodium channel (ENaC) by the cysteine protease cathepsin-S. Pflugers Arch. 464, 353–365. doi: 10.1007/s00424-012-1138-3, PMID: 22864553 PMC3448907

[ref84] Hammer G. D. Krylova I. Zhang Y. Darimont B. D. Simpson K. Weigel N. L.. (1999). Phosphorylation of the nuclear receptor SF-1 modulates cofactor recruitment: integration of hormone signaling in reproduction and stress. Mol. Cell 3, 521–526. 10230405 10.1016/s1097-2765(00)80480-3

[ref85] Hasegawa T. Zhao L. Caron K. M. Majdic G. Suzuki T. Shizawa S.. (2000). Developmental roles of the steroidogenic acute regulatory protein (StAR) as revealed by StAR knockout mice. Mol. Endocrinol. 14, 1462–1471. doi: 10.1210/mend.14.9.0515, PMID: 10976923

[ref86] He B. J. Anderson M. E. (2013). Aldosterone and cardiovascular disease: the heart of the matter. Trends Endocrinol. Metab. 24, 21–30. doi: 10.1016/j.tem.2012.09.004, PMID: 23040074 PMC3532553

[ref87] Heitzmann D. Derand R. Jungbauer S. Bandulik S. Sterner C. Schweda F.. (2008). Invalidation of TASK1 potassium channels disrupts adrenal gland zonation and mineralocorticoid homeostasis. EMBO J. 27, 179–187. doi: 10.1038/sj.emboj.7601934, PMID: 18034154 PMC2206116

[ref88] Hilfenhaus M. (1976). Circadian rhythm of the renin-angiotensin-aldosterone system in the rat. Arch. Toxicol. 36, 305–316. 1036902 10.1007/BF00340536

[ref89] Hirohama D. Ayuzawa N. Ueda K. Nishimoto M. Kawarazaki W. Watanabe A.. (2018). Aldosterone is essential for angiotensin II-induced upregulation of pendrin. J. Am. Soc. Nephrol. 29, 57–68. doi: 10.1681/ASN.2017030243, PMID: 29021385 PMC5748905

[ref90] Hodges R. R. Horikawa Y. Rios J. D. Shatos M. A. Dartt D. A. (2007). Effect of protein kinase C and ca(2+) on p42/p44 MAPK, Pyk2, and Src activation in rat conjunctival goblet cells. Exp. Eye Res. 85, 836–844. doi: 10.1016/j.exer.2007.08.019, PMID: 17919561 PMC2277506

[ref91] Holland O. B. Carr B. (1993). Modulation of aldosterone synthase messenger ribonucleic acid levels by dietary sodium and potassium and by adrenocorticotropin. Endocrinology 132, 2666–2673. 8389287 10.1210/endo.132.6.8389287

[ref92] Horvath A. Szabadkai G. Varnai P. Aranyi T. Wollheim C. B. Spat A.. (1998). Voltage dependent calcium channels in adrenal glomerulosa cells and in insulin producing cells. Cell Calcium 23, 33–42. 9570008 10.1016/s0143-4160(98)90072-0

[ref93] Huby A. C. Antonova G. Groenendyk J. Gomez-Sanchez C. E. Bollag W. B. Filosa J. A.. (2015). Adipocyte-derived hormone leptin is a direct regulator of aldosterone secretion, which promotes endothelial dysfunction and cardiac fibrosis. Circulation 132, 2134–2145. doi: 10.1161/CIRCULATIONAHA.115.018226, PMID: 26362633

[ref94] Huby A. C. Otvos L. Jr. Belin de Chantemele E. J. (2016). Leptin induces hypertension and endothelial dysfunction via aldosterone-dependent mechanisms in obese female mice. Hypertension 67, 1020–1028. doi: 10.1161/HYPERTENSIONAHA.115.06642, PMID: 26953321 PMC5088432

[ref95] Hughey R. P. Bruns J. B. Kinlough C. L. Harkleroad K. L. Tong Q. Carattino M. D.. (2004). Epithelial sodium channels are activated by furin-dependent proteolysis. J. Biol. Chem. 279, 18111–18114. doi: 10.1074/jbc.C400080200, PMID: 15007080

[ref96] Hume R. Kelly R. W. Taylor P. L. Boyd G. S. (1984). The catalytic cycle of cytochrome P-450scc and intermediates in the conversion of cholesterol to pregnenolone. Eur. J. Biochem. 140, 583–591. 6723652 10.1111/j.1432-1033.1984.tb08142.x

[ref97] Ishihara K. Yamamoto T. Kubo Y. (2009). Heteromeric assembly of inward rectifier channel subunit Kir2.1 with Kir3.1 and with Kir3.4. Biochem. Biophys. Res. Commun. 380, 832–837. doi: 10.1016/j.bbrc.2009.01.179, PMID: 19338762

[ref98] Ishimura K. Fujita H. (1997). Light and electron microscopic immunohistochemistry of the localization of adrenal steroidogenic enzymes. Microsc. Res. Tech. 36, 445–453. 9142691 10.1002/(SICI)1097-0029(19970315)36:6<445::AID-JEMT2>3.0.CO;2-H

[ref99] Itoh S. Abe K. Nushiro N. Omata K. Yasujima M. Yoshinaga K. (1987). Effect of atrial natriuretic factor on renin release in isolated afferent arterioles. Kidney Int. 32, 493–497. 2963164 10.1038/ki.1987.237

[ref100] Ji A. X. Prive G. G. (2013). Crystal structure of KLHL3 in complex with Cullin3. PLoS One 8:e60445. doi: 10.1371/journal.pone.0060445, PMID: 23573258 PMC3616122

[ref101] Jo Y. King S. R. Khan S. A. Stocco D. M. (2005). Involvement of protein kinase C and cyclic adenosine 3',5'-monophosphate-dependent kinase in steroidogenic acute regulatory protein expression and steroid biosynthesis in Leydig cells. Biol. Reprod. 73, 244–255. doi: 10.1095/biolreprod.104.037721, PMID: 15814901

[ref102] Kanazirska M. V. Vassilev P. M. Quinn S. J. Tillotson D. L. Williams G. H. (1992). Single K+ channels in adrenal zona glomerulosa cells. II. Inhibition by angiotensin II. Am. J. Phys. 263, E760–E765. 10.1152/ajpendo.1992.263.4.E760 1415697

[ref103] Kapas S. Purbrick A. Hinson J. P. (1995). Role of tyrosine kinase and protein kinase C in the steroidogenic actions of angiotensin II, alpha-melanocyte-stimulating hormone and corticotropin in the rat adrenal cortex. Biochem. J. 305(Pt 2), 433–438. 7832756 10.1042/bj3050433 PMC1136380

[ref104] Kashlan O. B. Kleyman T. R. (2011). ENaC structure and function in the wake of a resolved structure of a family member. Am. J. Physiol. Renal Physiol. 301, F684–F696. doi: 10.1152/ajprenal.00259.2011, PMID: 21753073 PMC3191808

[ref105] Kayes-Wandover K. M. White P. C. (2000). Steroidogenic enzyme gene expression in the human heart. J. Clin. Endocrinol. Metab. 85, 2519–2525. doi: 10.1210/jc.85.7.2519, PMID: 10902803

[ref106] Kem D. C. Weinberger M. H. Gomez-Sanchez C. Kramer N. J. Lerman R. Furuyama S.. (1973). Circadian rhythm of plasma aldosterone concentration in patients with primary aldosteronism. J. Clin. Invest. 52, 2272–2277. 4353776 10.1172/JCI107414 PMC333030

[ref107] Kleyman T. R. Eaton D. C. (2020). Regulating ENaC’s gate. Am. J. Physiol. Cell Physiol. 318, C150–C162. doi: 10.1152/ajpcell.00418.2019, PMID: 31721612 PMC6985836

[ref108] Kurukulasuriya L. R. Stas S. Lastra G. Manrique C. Sowers J. R. (2011). Hypertension in obesity. Med. Clin. North Am. 95, 903–917. doi: 10.1016/j.mcna.2011.06.004 21855699

[ref109] Kyossev Z. Walker P. D. Reeves W. B. (1996). Immunolocalization of NAD-dependent 11 beta-hydroxysteroid dehydrogenase in human kidney and colon. Kidney Int. 49, 271–281. 8770980 10.1038/ki.1996.39

[ref110] Lamarre-Cliche M. de Champlain J. Lacourciere Y. Poirier L. Karas M. Larochelle P. (2005). Effects of circadian rhythms, posture, and medication on renin-aldosterone interrelations in essential hypertensives. Am. J. Hypertens. 18, 56–64. doi: 10.1016/j.amjhyper.2004.08.025, PMID: 15691618

[ref111] Le T. Schimmer B. P. (2001). The regulation of MAPKs in Y1 mouse adrenocortical tumor cells. Endocrinology 142, 4282–4287. doi: 10.1210/endo.142.10.8441, PMID: 11564685

[ref112] Li H. Yu X. Cicala M. V. Mantero F. Benbrook A. Veitla V.. (2015). Prevalence of angiotensin II type 1 receptor (AT1R)-activating autoantibodies in primary aldosteronism. J. Am. Soc. Hypertens. 9, 15–20. doi: 10.1016/j.jash.2014.10.009, PMID: 25537460 PMC4314451

[ref113] Loffing J. Kaissling B. (2003). Sodium and calcium transport pathways along the mammalian distal nephron: from rabbit to human. Am. J. Physiol. Renal Physiol. 284, F628–F643. doi: 10.1152/ajprenal.00217.2002, PMID: 12620920

[ref114] Lopez-Cayuqueo K. I. Chavez-Canales M. Pillot A. Houillier P. Jayat M. Baraka-Vidot J.. (2018). A mouse model of pseudohypoaldosteronism type II reveals a novel mechanism of renal tubular acidosis. Kidney Int. 94, 514–523. doi: 10.1016/j.kint.2018.05.001, PMID: 30146013

[ref115] Louis-Dit-Picard H. Barc J. Trujillano D. Miserey-Lenkei S. Bouatia-Naji N. Pylypenko O.. (2012). KLHL3 mutations cause familial hyperkalemic hypertension by impairing ion transport in the distal nephron. Nat. Genet. 44, 456–460, S1–3. doi: 10.1038/ng.2218, PMID: 22406640

[ref116] Lu M. Wang J. Jones K. T. Ives H. E. Feldman M. E. Yao L. J.. (2010). mTOR complex-2 activates ENaC by phosphorylating SGK1. J. Am. Soc. Nephrol. 21, 811–818. doi: 10.1681/ASN.2009111168, PMID: 20338997 PMC2865740

[ref117] Lubarski I. Pihakaski-Maunsbach K. Karlish S. J. Maunsbach A. B. Garty H. (2005). Interaction with the Na, K-ATPase and tissue distribution of FXYD5 (related to ion channel). J. Biol. Chem. 280, 37717–37724. doi: 10.1074/jbc.M506397200, PMID: 16148001

[ref118] Lymangrover J. R. Matthews E. K. Saffran M. (1982). Membrane potential changes of mouse adrenal zona fasciculata cells in response to adrenocorticotropin and adenosine 3′,5′-monophosphate. Endocrinology 110, 462–468. 6276136 10.1210/endo-110-2-462

[ref119] Maack T. Marion D. N. Camargo M. J. Kleinert H. D. Laragh J. H. Vaughan E. D. Jr.. (1984). Effects of auriculin (atrial natriuretic factor) on blood pressure, renal function, and the renin-aldosterone system in dogs. Am. J. Med. 77, 1069–1075. 6239544 10.1016/0002-9343(84)90190-6

[ref120] Manna P. R. Huhtaniemi I. T. Stocco D. M. (2009). Mechanisms of protein kinase C signaling in the modulation of 3′,5′-cyclic adenosine monophosphate-mediated steroidogenesis in mouse gonadal cells. Endocrinology 150, 3308–3317. doi: 10.1210/en.2008-1668 19282384 PMC2703526

[ref121] Martinez-Rumayor A. Richards A. M. Burnett J. C. Januzzi J. L. Jr. (2008). Biology of the natriuretic peptides. Am. J. Cardiol. 101, 3–8. doi: 10.1016/j.amjcard.2007.11.012, PMID: 18243856

[ref122] Mitani F. Miyamoto H. Mukai K. Ishimura Y. (1996). Effects of long term stimulation of ACTH and angiotensin II-secretions on the rat adrenal cortex. Endocr. Res. 22, 421–431. 8969893 10.1080/07435809609043728

[ref123] Monticone S. Hattangady N. G. Nishimoto K. Mantero F. Rubin B. Cicala M. V.. (2012). Effect of KCNJ5 mutations on gene expression in aldosterone-producing adenomas and adrenocortical cells. J. Clin. Endocrinol. Metab. 97, E1567–E1572. doi: 10.1210/jc.2011-3132, PMID: 22628608 PMC3410264

[ref124] Mori Y. Wakabayashi M. Mori T. Araki Y. Sohara E. Rai T.. (2013). Decrease of WNK4 ubiquitination by disease-causing mutations of KLHL3 through different molecular mechanisms. Biochem. Biophys. Res. Commun. 439, 30–34. doi: 10.1016/j.bbrc.2013.08.035, PMID: 23962426

[ref125] Mulatero P. Tauber P. Zennaro M. C. Monticone S. Lang K. Beuschlein F.. (2012). KCNJ5 mutations in European families with nonglucocorticoid remediable familial hyperaldosteronism. Hypertension 59, 235–240. doi: 10.1161/HYPERTENSIONAHA.111.183996, PMID: 22203740

[ref126] Na T. Wu G. Zhang W. Dong W. J. Peng J. B. (2013). Disease-causing R1185C mutation of WNK4 disrupts a regulatory mechanism involving calmodulin binding and SGK1 phosphorylation sites. Am. J. Physiol. Renal Physiol. 304, F8–F18. doi: 10.1152/ajprenal.00284.2012, PMID: 23054253 PMC3543615

[ref127] Nagase M. Yoshida S. Shibata S. Nagase T. Gotoda T. Ando K.. (2006). Enhanced aldosterone signaling in the early nephropathy of rats with metabolic syndrome: possible contribution of fat-derived factors. J. Am. Soc. Nephrol. 17, 3438–3446. doi: 10.1681/ASN.2006080944, PMID: 17082236

[ref128] Nanba K. Blinder A. R. Rege J. Hattangady N. G. Else T. Liu C. J.. (2020). Somatic CACNA1H mutation as a cause of aldosterone-producing adenoma. Hypertension 75, 645–649. doi: 10.1161/HYPERTENSIONAHA.119.14349, PMID: 31983310 PMC7059016

[ref129] Nanba K. Chen A. Nishimoto K. Rainey W. E. (2015). Role of ca(2+)/calmodulin-dependent protein kinase kinase in adrenal aldosterone production. Endocrinology 156, 1750–1756. doi: 10.1210/en.2014-1782, PMID: 25679868 PMC4398758

[ref130] Naray-Fejes-Toth A. Rusvai E. Fejes-Toth G. (1994). Minealocorticoid receptors and 11 beta-steroid dehydrogenase activity in renal principal and intercalated cells. Am. J. Phys. 266, F76–F80. 10.1152/ajprenal.1994.266.1.F76 8304486

[ref131] Naray-Fejes-Toth A. Snyder P. M. Fejes-Toth G. (2004). The kidney-specific WNK1 isoform is induced by aldosterone and stimulates epithelial sodium channel-mediated Na+ transport. Proc. Natl. Acad. Sci. U. S. A. 101, 17434–17439. doi: 10.1073/pnas.0408146101, PMID: 15583131 PMC536044

[ref132] Natarajan R. Dunn W. D. Stern N. Nadler J. (1990). Key role of diacylglycerol-mediated 12-lipoxygenase product formation in angiotensin II-induced aldosterone synthesis. Mol. Cell. Endocrinol. 72, 73–80. 2178102 10.1016/0303-7207(90)90096-q

[ref133] Natarajan R. Stern N. Hsueh W. Do Y. Nadler J. (1988a). Role of the lipoxygenase pathway in angiotensin II-mediated aldosterone biosynthesis in human adrenal glomerulosa cells. J. Clin. Endocrinol. Metab. 67, 584–591. 2842363 10.1210/jcem-67-3-584

[ref134] Natarajan R. Stern N. Nadler J. (1988b). Diacylglycerol provides arachidonic acid for lipoxygenase products that mediate angiotensin II-induced aldosterone synthesis. Biochem. Biophys. Res. Commun. 156, 717–724. 2847716 10.1016/s0006-291x(88)80902-1

[ref135] Nguyen A. T. Zhang Y. (2011). The diverse functions of Dot1 and H3K79 methylation. Genes Dev. 25, 1345–1358. doi: 10.1101/gad.2057811, PMID: 21724828 PMC3134078

[ref136] Nogueira E. F. Rainey W. E. (2010). Regulation of aldosterone synthase by activator transcription factor/cAMP response element-binding protein family members. Endocrinology 151, 1060–1070. doi: 10.1210/en.2009-0977, PMID: 20097716 PMC2840695

[ref137] Noreng S. Bharadwaj A. Posert R. Yoshioka C. Baconguis I. (2018). Structure of the human epithelial sodium channel by cryo-electron microscopy. elife 7:e39340. doi: 10.7554/eLife.39340, PMID: 30251954 PMC6197857

[ref138] Ohnishi T. Wada A. Lauber M. Yamano T. Okamoto M. (1988). Aldosterone biosynthesis in mitochondria of isolated zones of adrenal cortex. J. Steroid Biochem. 31, 73–81. 3398531 10.1016/0022-4731(88)90208-7

[ref139] Ohta A. Schumacher F. R. Mehellou Y. Johnson C. Knebel A. Macartney T. J.. (2013). The CUL3-KLHL3 E3 ligase complex mutated in Gordon's hypertension syndrome interacts with and ubiquitylates WNK isoforms: disease-causing mutations in KLHL3 and WNK4 disrupt interaction. Biochem. J. 451, 111–122. doi: 10.1042/BJ20121903, PMID: 23387299 PMC3632089

[ref140] Oki K. Plonczynski M. W. Lam M. L. Gomez-Sanchez E. P. Gomez-Sanchez C. E. (2012a). The potassium channel, Kir3.4 participates in angiotensin II-stimulated aldosterone production by a human adrenocortical cell line. Endocrinology 153, 4328–4335. doi: 10.1210/en.2012-1241, PMID: 22798349 PMC3423613

[ref141] Oki K. Plonczynski M. W. Luis Lam M. Gomez-Sanchez E. P. Gomez-Sanchez C. E. (2012b). Potassium channel mutant KCNJ5 T158A expression in HAC-15 cells increases aldosterone synthesis. Endocrinology 153, 1774–1782. doi: 10.1210/en.2011-1733, PMID: 22315453 PMC3320257

[ref142] Okubo S. Niimura F. Nishimura H. Takemoto F. Fogo A. Matsusaka T.. (1997). Angiotensin-independent mechanism for aldosterone synthesis during chronic extracellular fluid volume depletion. J. Clin. Invest. 99, 855–860. 9062342 10.1172/JCI119249 PMC507892

[ref143] Olala L. O. Choudhary V. Johnson M. H. Bollag W. B. (2014). Angiotensin II-induced protein kinase D activates the ATF/CREB family of transcription factors and promotes StAR mRNA expression. Endocrinology 155, 2524–2533. doi: 10.1210/en.2013-1485, PMID: 24708239 PMC4060184

[ref144] Olejnik A. Franczak A. Krzywonos-Zawadzka A. Kaluzna-Oleksy M. Bil-Lula I. (2018). The biological role of klotho protein in the development of cardiovascular diseases. Biomed. Res. Int. 2018:5171945. doi: 10.1155/2018/5171945 30671457 PMC6323445

[ref145] Ouyang J. Hu D. Wang B. Shi T. Ma X. Li H.. (2011). Differential effects of down-regulated steroidogenic factor-1 on basal and angiotensin II-induced aldosterone secretion. J. Endocrinol. Investig. 34, 671–675. doi: 10.3275/7413, PMID: 21169726

[ref146] Pan Y. J. Young D. B. (1982). Experimental aldosterone hypertension in the dog. Hypertension 4, 279–287. 7040227 10.1161/01.hyp.4.2.279

[ref147] Papadopoulou-Marketou N. Vaidya A. Dluhy R. Chrousos G. P. (2000). “Hyperaldosteronism,” in Endotext (South Dartmouth, MA: Endotext).

[ref148] Park J. Leong M. L. Buse P. Maiyar A. C. Firestone G. L. Hemmings B. A. (1999). Serum and glucocorticoid-inducible kinase (SGK) is a target of the PI 3-kinase-stimulated signaling pathway. EMBO J. 18, 3024–3033. 10357815 10.1093/emboj/18.11.3024 PMC1171384

[ref149] Peti-Peterdi J. Harris R. C. (2010). Macula densa sensing and signaling mechanisms of renin release. J. Am. Soc. Nephrol. 21, 1093–1096. doi: 10.1681/ASN.2009070759, PMID: 20360309 PMC4577295

[ref150] Piazza M. Seccia T. M. Caroccia B. Rossitto G. Scarpa R. Persichitti P.. (2019). AT1AA (angiotensin II Type-1 receptor autoantibodies): cause or consequence of human primary aldosteronism? Hypertension 74, 793–799. doi: 10.1161/HYPERTENSIONAHA.119.13388, PMID: 31476908

[ref151] Pilon A. Martin G. Bultel-Brienne S. Junquero D. Delhon A. Fruchart J. C.. (2003). Regulation of the scavenger receptor BI and the LDL receptor by activators of aldosterone production, angiotensin II and PMA, in the human NCI-H295R adrenocortical cell line. Biochim. Biophys. Acta 1631, 218–228. doi: 10.1016/S1388-1981(03)00020-9, PMID: 12668173

[ref152] Qian J. Zhong J. Yan M. Cheng P. Shi H. Hao C.. (2018). Circulating alpha-klotho is related to plasma aldosterone and its follow-up change predicts CKD progression. Kidney Blood Press. Res. 43, 836–846. doi: 10.1159/000490138, PMID: 29843135

[ref153] Quinn S. J. Cornwall M. C. Williams G. H. (1987). Electrical properties of isolated rat adrenal glomerulosa and fasciculata cells. Endocrinology 120, 903–914. 3803318 10.1210/endo-120-3-903

[ref154] Rashmi P. Colussi G. Ng M. Wu X. Kidwai A. Pearce D. (2017). Glucocorticoid-induced leucine zipper protein regulates sodium and potassium balance in the distal nephron. Kidney Int. 91, 1159–1177. doi: 10.1016/j.kint.2016.10.038, PMID: 28094030 PMC6034684

[ref155] Reilly R. F. Ellison D. H. (2000). Mammalian distal tubule: physiology, pathophysiology, and molecular anatomy. Physiol. Rev. 80, 277–313. doi: 10.1152/physrev.2000.80.1.277, PMID: 10617770

[ref156] Reimer E. N. Walenda G. Seidel E. Scholl U. I. (2016). CACNA1H(M1549V) mutant calcium channel causes autonomous aldosterone production in HAC15 cells and is inhibited by Mibefradil. Endocrinology 157, 3016–3022. doi: 10.1210/en.2016-1170, PMID: 27258646

[ref157] Reisenauer M. R. Anderson M. Huang L. Zhang Z. Zhou Q. Kone B. C.. (2009). AF17 competes with AF9 for binding to Dot1a to up-regulate transcription of epithelial Na+ channel alpha. J. Biol. Chem. 284, 35659–35669. doi: 10.1074/jbc.M109.038448, PMID: 19864429 PMC2790997

[ref158] Richards J. Jeffers L. A. All S. C. Cheng K. Y. Gumz M. L. (2013). Role of Per1 and the mineralocorticoid receptor in the coordinate regulation of alphaENaC in renal cortical collecting duct cells. Front. Physiol. 4:253. doi: 10.3389/fphys.2013.00253 24062694 PMC3775537

[ref159] Ring A. M. Cheng S. X. Leng Q. Kahle K. T. Rinehart J. Lalioti M. D.. (2007a). WNK4 regulates activity of the epithelial Na+ channel in vitro and in vivo. Proc. Natl. Acad. Sci. U. S. A. 104, 4020–4024. doi: 10.1073/pnas.0611727104, PMID: 17360470 PMC1805455

[ref160] Ring A. M. Leng Q. Rinehart J. Wilson F. H. Kahle K. T. Hebert S. C.. (2007b). An SGK1 site in WNK4 regulates Na+ channel and K+ channel activity and has implications for aldosterone signaling and K+ homeostasis. Proc. Natl. Acad. Sci. U. S. A. 104, 4025–4029. doi: 10.1073/pnas.0611728104, PMID: 17360471 PMC1803763

[ref161] Rocha R. Funder J. W. (2002). The pathophysiology of aldosterone in the cardiovascular system. Ann. N. Y. Acad. Sci. 970, 89–100. doi: 10.1111/j.1749-6632.2002.tb04415.x 12381544

[ref162] Ronti T. Lupattelli G. Mannarino E. (2006). The endocrine function of adipose tissue: an update. Clin. Endocrinol. 64, 355–365. doi: 10.1111/j.1365-2265.2006.02474.x, PMID: 16584505

[ref163] Rossier M. F. Ertel E. A. Vallotton M. B. Capponi A. M. (1998). Inhibitory action of mibefradil on calcium signaling and aldosterone synthesis in bovine adrenal glomerulosa cells. J. Pharmacol. Exp. Ther. 287, 824–831. 9864260

[ref164] Rossier B. C. Stutts M. J. (2009). Activation of the epithelial sodium channel (ENaC) by serine proteases. Annu. Rev. Physiol. 71, 361–379. doi: 10.1146/annurev.physiol.010908.163108 18928407

[ref165] Rossitto G. Regolisti G. Rossi E. Negro A. Nicoli D. Casali B.. (2013). Elevation of angiotensin-II type-1-receptor autoantibodies titer in primary aldosteronism as a result of aldosterone-producing adenoma. Hypertension 61, 526–533. doi: 10.1161/HYPERTENSIONAHA.112.202945, PMID: 23248149

[ref166] Rotin D. (2008). Role of the UPS in Liddle syndrome. BMC Biochem. 9(Suppl 1):S5. doi: 10.1186/1471-2091-9-S1-S5, PMID: 19007435 PMC2582799

[ref167] Roy A. Al-bataineh M. M. Pastor-Soler N. M. (2015). Collecting duct intercalated cell function and regulation. Clin. J. Am. Soc. Nephrol. 10, 305–324. doi: 10.2215/CJN.08880914 25632105 PMC4317747

[ref168] Sackmann S. Lichtenauer U. Shapiro I. Reincke M. Beuschlein F. (2011). Aldosterone producing adrenal adenomas are characterized by activation of calcium/calmodulin-dependent protein kinase (CaMK) dependent pathways. Horm. Metab. Res. 43, 106–111. doi: 10.1055/s-0030-1269899, PMID: 21249615

[ref169] Sadovsky Y. Crawford P. A. Woodson K. G. Polish J. A. Clements M. A. Tourtellotte L. M.. (1995). Mice deficient in the orphan receptor steroidogenic factor 1 lack adrenal glands and gonads but express P450 side-chain-cleavage enzyme in the placenta and have normal embryonic serum levels of corticosteroids. Proc. Natl. Acad. Sci. U. S. A. 92, 10939–10943. 7479914 10.1073/pnas.92.24.10939 PMC40546

[ref170] Scholl U. I. Goh G. Stolting G. de Oliveira R. C. Choi M. Overton J. D.. (2013). Somatic and germline CACNA1D calcium channel mutations in aldosterone-producing adenomas and primary aldosteronism. Nat. Genet. 45, 1050–1054. doi: 10.1038/ng.2695, PMID: 23913001 PMC3876926

[ref171] Scholl U. I. Nelson-Williams C. Yue P. Grekin R. Wyatt R. J. Dillon M. J.. (2012). Hypertension with or without adrenal hyperplasia due to different inherited mutations in the potassium channel KCNJ5. Proc. Natl. Acad. Sci. U. S. A. 109, 2533–2538. doi: 10.1073/pnas.1121407109, PMID: 22308486 PMC3289329

[ref172] Scholl U. I. Stolting G. Nelson-Williams C. Vichot A. A. Choi M. Loring E.. (2015). Recurrent gain of function mutation in calcium channel CACNA1H causes early-onset hypertension with primary aldosteronism. elife 4:e06315. doi: 10.7554/eLife.06315, PMID: 25907736 PMC4408447

[ref173] Sculptoreanu A. Scheuer T. Catterall W. A. (1993). Voltage-dependent potentiation of L-type Ca^2+^ channels due to phosphorylation by cAMP-dependent protein kinase. Nature 364, 240–243. 8391648 10.1038/364240a0

[ref174] Seely E. W. Conlin P. R. Brent G. A. Dluhy R. G. (1989). Adrenocorticotropin stimulation of aldosterone: prolonged continuous versus pulsatile infusion. J. Clin. Endocrinol. Metab. 69, 1028–1032. 2551915 10.1210/jcem-69-5-1028

[ref175] Sewer M. B. Li D. (2008). Regulation of steroid hormone biosynthesis by the cytoskeleton. Lipids 43, 1109–1115. doi: 10.1007/s11745-008-3221-2, PMID: 18726632 PMC2717900

[ref176] Sewer M. B. Waterman M. R. (2003). CAMP-dependent protein kinase enhances CYP17 transcription via MKP-1 activation in H295R human adrenocortical cells. J. Biol. Chem. 278, 8106–8111. doi: 10.1074/jbc.M210264200, PMID: 12506119

[ref177] Shibata S. Fujita T. (2011). The kidneys and aldosterone/mineralocorticoid receptor system in salt-sensitive hypertension. Curr. Hypertens. Rep. 13, 109–115. doi: 10.1007/s11906-010-0175-6, PMID: 21207253 PMC3047054

[ref178] Shibata S. Rinehart J. Zhang J. Moeckel G. Castaneda-Bueno M. Stiegler A. L.. (2013a). Mineralocorticoid receptor phosphorylation regulates ligand binding and renal response to volume depletion and hyperkalemia. Cell Metab. 18, 660–671. doi: 10.1016/j.cmet.2013.10.005, PMID: 24206662 PMC3909709

[ref179] Shibata S. Zhang J. Puthumana J. Stone K. L. Lifton R. P. (2013b). Kelch-like 3 and Cullin 3 regulate electrolyte homeostasis via ubiquitination and degradation of WNK4. Proc. Natl. Acad. Sci. U. S. A. 110, 7838–7843. doi: 10.1073/pnas.1304592110, PMID: 23576762 PMC3651502

[ref180] Shimkets R. A. Warnock D. G. Bositis C. M. Nelson-Williams C. Hansson J. H. Schambelan M.. (1994). Liddle’s syndrome: heritable human hypertension caused by mutations in the beta subunit of the epithelial sodium channel. Cell 79, 407–414. 7954808 10.1016/0092-8674(94)90250-x

[ref181] Simpson S. A. Tait J. F. Wettstein A. Neher R. Von Euw J. Schindler O.. (1954). Constitution of aldosterone, a new mineralocorticoid. Experientia 10, 132–133. doi: 10.1007/BF02158515, PMID: 13161890

[ref182] Solocinski K. Holzworth M. Wen X. Cheng K. Y. Lynch I. J. Cain B. D.. (2017). Desoxycorticosterone pivalate-salt treatment leads to non-dipping hypertension in Per1 knockout mice. Acta Physiol. 220, 72–82. doi: 10.1111/apha.12804, PMID: 27636900 PMC5354999

[ref183] Sonoyama T. Sone M. Tamura N. Honda K. Taura D. Kojima K.. (2014). Role of endogenous ACTH on circadian aldosterone rhythm in patients with primary aldosteronism. Endocr. Connect. 3, 173–179. doi: 10.1530/EC-14-0086, PMID: 25239966 PMC4168680

[ref184] Soundararajan R. Wang J. Melters D. Pearce D. (2010). Glucocorticoid-induced leucine zipper 1 stimulates the epithelial sodium channel by regulating serum- and glucocorticoid-induced kinase 1 stability and subcellular localization. J. Biol. Chem. 285, 39905–39913. doi: 10.1074/jbc.M110.161133, PMID: 20947508 PMC3000972

[ref185] Soundararajan R. Zhang T. T. Wang J. Vandewalle A. Pearce D. (2005). A novel role for glucocorticoid-induced leucine zipper protein in epithelial sodium channel-mediated sodium transport. J. Biol. Chem. 280, 39970–39981. doi: 10.1074/jbc.M508658200, PMID: 16216878

[ref186] Spat A. Enyedi P. Hajnoczky G. Hunyady L. (1991). Generation and role of calcium signal in adrenal glomerulosa cells. Exp. Physiol. 76, 859–885. 1662965 10.1113/expphysiol.1991.sp003550

[ref187] Staruschenko A. Adams E. Booth R. E. Stockand J. D. (2005). Epithelial Na+ channel subunit stoichiometry. Biophys. J. 88, 3966–3975. doi: 10.1529/biophysj.104.056804, PMID: 15821171 PMC1305628

[ref188] Staub O. Dho S. Henry P. Correa J. Ishikawa T. McGlade J.. (1996). WW domains of Nedd4 bind to the proline-rich PY motifs in the epithelial Na+ channel deleted in Liddle’s syndrome. EMBO J. 15, 2371–2380. 8665844 PMC450167

[ref189] Steckelings U. M. Rompe F. Kaschina E. Namsolleck P. Grzesiak A. Funke-Kaiser H.. (2010). The past, present and future of angiotensin II type 2 receptor stimulation. J. Renin-Angiotensin-Aldosterone Syst. 11, 67–73. doi: 10.1177/1470320309347791, PMID: 19861348

[ref190] Stern N. Sowers J. R. McGinty D. Beahm E. Littner M. Catania R.. (1986). Circadian rhythm of plasma renin activity in older normal and essential hypertensive men: relation with inactive renin, aldosterone, cortisol and REM sleep. J. Hypertens. 4, 543–550. 3540117 10.1097/00004872-198610000-00005

[ref191] Susa K. Sohara E. Rai T. Zeniya M. Mori Y. Mori T.. (2014). Impaired degradation of WNK1 and WNK4 kinases causes PHAII in mutant KLHL3 knock-in mice. Hum. Mol. Genet. 23, 5052–5060. doi: 10.1093/hmg/ddu217, PMID: 24821705

[ref192] Takagi M. Takagi M. Franco-Saenz R. Mulrow P. J. (1988). Effect of atrial natriuretic peptide on renin release in a superfusion system of kidney slices and dispersed juxtaglomerular cells. Endocrinology 122, 1437–1442. 2831030 10.1210/endo-122-4-1437

[ref193] Takeda R. Miyamori I. Ikeda M. Koshida H. Takeda Y. Yasuhara S.. (1984). Circadian rhythm of plasma aldosterone and time dependent alterations of aldosterone regulators. J. Steroid Biochem. 20, 321–323. 6323866 10.1016/0022-4731(84)90225-5

[ref194] Takeda Y. Yoneda T. Demura M. Miyamori I. Mabuchi H. (2000). Cardiac aldosterone production in genetically hypertensive rats. Hypertension 36, 495–500. doi: 10.1161/01.HYP.36.4.495, PMID: 11040225

[ref195] Takemoto-Kimura S. Suzuki K. Horigane S. I. Kamijo S. Inoue M. Sakamoto M.. (2017). Calmodulin kinases: essential regulators in health and disease. J. Neurochem. 141, 808–818. doi: 10.1111/jnc.14020, PMID: 28295333

[ref196] Taylor C. W. Thorn P. (2001). Calcium signalling: IP3 rises again…and again. Curr. Biol. 11, R352–R355. doi: 10.1016/S0960-9822(01)00192-0, PMID: 11369246

[ref197] Teng-umnuay P. Verlander J. W. Yuan W. Tisher C. C. Madsen K. M. (1996). Identification of distinct subpopulations of intercalated cells in the mouse collecting duct. J. Am. Soc. Nephrol. 7, 260–274. 8785396 10.1681/ASN.V72260

[ref198] Thosar S. S. Rueda J. F. Berman A. M. Lasarev M. R. Herzig M. X. Clemons N. A.. (2019). Separate and interacting effects of the endogenous circadian system and behaviors on plasma aldosterone in humans. Am. J. Physiol. Regul. Integr. Comp. Physiol. 316, R157–R164. doi: 10.1152/ajpregu.00314.2018, PMID: 30521366 PMC6397357

[ref199] Tokumitsu H. Enslen H. Soderling T. R. (1995). Characterization of a Ca^2+^/calmodulin-dependent protein kinase cascade. Molecular cloning and expression of calcium/calmodulin-dependent protein kinase kinase. J. Biol. Chem. 270, 19320–19324. 7642608 10.1074/jbc.270.33.19320

[ref200] Tremblay A. LeHoux J. G. (1993). Transcriptional activation of adrenocortical steroidogenic genes by high potassium or low sodium intake. FEBS Lett. 317, 211–215. 7678819 10.1016/0014-5793(93)81278-8

[ref201] Tsuruoka S. Schwartz G. J. (1999). Mechanisms of HCO(−)(3) secretion in the rabbit connecting segment. Am. J. Phys. 277, F567–F574. 10.1152/ajprenal.1999.277.4.F567 10516281

[ref202] Uebele V. N. Nuss C. E. Renger J. J. Connolly T. M. (2004). Role of voltage-gated calcium channels in potassium-stimulated aldosterone secretion from rat adrenal zona glomerulosa cells. J. Steroid Biochem. Mol. Biol. 92, 209–218. doi: 10.1016/j.jsbmb.2004.04.012, PMID: 15555914

[ref203] Varnai P. Osipenko O. N. Vizi E. S. Spat A. (1995). Activation of calcium current in voltage-clamped rat glomerulosa cells by potassium ions. J. Physiol. 483(Pt 1), 67–78. 7776242 10.1113/jphysiol.1995.sp020568 PMC1157872

[ref204] Varnai P. Petheo G. L. Makara J. K. Spat A. (1998). Electrophysiological study on the high K+ sensitivity of rat glomerulosa cells. Pflugers Arch. 435, 429–431. 9426301 10.1007/s004240050534

[ref205] Verlander J. W. Madsen K. M. Cannon J. K. Tisher C. C. (1994). Activation of acid-secreting intercalated cells in rabbit collecting duct with ammonium chloride loading. Am. J. Phys. 266, F633–F645. 10.1152/ajprenal.1994.266.4.F633 8184897

[ref206] Verrey F. Fakitsas P. Adam G. Staub O. (2008). Early transcriptional control of ENaC (de)ubiquitylation by aldosterone. Kidney Int. 73, 691–696. doi: 10.1038/sj.ki.5002737, PMID: 18094676

[ref207] Vinson G. P. (2016). Functional zonation of the adult mammalian adrenal cortex. Front. Neurosci. 10:238. doi: 10.3389/fnins.2016.00238 27378832 PMC4908136

[ref208] Wakabayashi M. Mori T. Isobe K. Sohara E. Susa K. Araki Y.. (2013). Impaired KLHL3-mediated ubiquitination of WNK4 causes human hypertension. Cell Rep. 3, 858–868. doi: 10.1016/j.celrep.2013.02.024, PMID: 23453970

[ref209] Wang Q. J. (2006). PKD at the crossroads of DAG and PKC signaling. Trends Pharmacol. Sci. 27, 317–323. doi: 10.1016/j.tips.2006.04.003, PMID: 16678913

[ref210] Wang J. J. Peng K. Y. Wu V. C. Tseng F. Y. Wu K. D. (2017). CTNNB1 mutation in aldosterone producing adenoma. Endocrinol. Metab. 32, 332–338. doi: 10.3803/EnM.2017.32.3.332, PMID: 28956362 PMC5620029

[ref211] Weidmann P. Hellmueller B. Uehlinger D. E. Lang R. E. Gnaedinger M. P. Hasler L.. (1986). Plasma levels and cardiovascular, endocrine, and excretory effects of atrial natriuretic peptide during different sodium intakes in man. J. Clin. Endocrinol. Metab. 62, 1027–1036. 2937799 10.1210/jcem-62-5-1027

[ref212] Williams T. A. Monticone S. Mulatero P. (2015). KCNJ5 mutations are the most frequent genetic alteration in primary aldosteronism. Hypertension 65, 507–509. doi: 10.1161/HYPERTENSIONAHA.114.04636 25624337

[ref213] Wilson F. H. Disse-Nicodeme S. Choate K. A. Ishikawa K. Nelson-Williams C. Desitter I.. (2001). Human hypertension caused by mutations in WNK kinases. Science 293, 1107–1112. doi: 10.1126/science.1062844 11498583

[ref214] Winnay J. N. Hammer G. D. (2006). Adrenocorticotropic hormone-mediated signaling cascades coordinate a cyclic pattern of steroidogenic factor 1-dependent transcriptional activation. Mol. Endocrinol. 20, 147–166. doi: 10.1210/me.2005-0215, PMID: 16109736

[ref215] Wu H. Chen L. Zhou Q. Zhang W. (2011). AF17 facilitates Dot1a nuclear export and upregulates ENaC-mediated Na+ transport in renal collecting duct cells. PLoS One 6:e27429. doi: 10.1371/journal.pone.0027429, PMID: 22087315 PMC3210795

[ref216] Wu G. Peng J. B. (2013). Disease-causing mutations in KLHL3 impair its effect on WNK4 degradation. FEBS Lett. 587, 1717–1722. doi: 10.1016/j.febslet.2013.04.032, PMID: 23665031 PMC3697765

[ref217] Xu B. E. Stippec S. Chu P. Y. Lazrak A. Li X. J. Lee B. H.. (2005a). WNK1 activates SGK1 to regulate the epithelial sodium channel. Proc. Natl. Acad. Sci. U. S. A. 102, 10315–10320. doi: 10.1073/pnas.0504422102, PMID: 16006511 PMC1177404

[ref218] Xu B. E. Stippec S. Lazrak A. Huang C. L. Cobb M. H. (2005b). WNK1 activates SGK1 by a phosphatidylinositol 3-kinase-dependent and non-catalytic mechanism. J. Biol. Chem. 280, 34218–34223. doi: 10.1074/jbc.M505735200, PMID: 16081417

[ref219] Yao J. Davies L. A. Howard J. D. Adney S. K. Welsby P. J. Howell N.. (2006). Molecular basis for the modulation of native T-type Ca^2+^ channels in vivo by Ca^2+^/calmodulin-dependent protein kinase II. J. Clin. Invest. 116, 2403–2412. doi: 10.1172/JCI27918, PMID: 16917542 PMC1550277

[ref220] Yoshimoto T. Hirata Y. (2007). Aldosterone as a cardiovascular risk hormone. Endocr. J. 54, 359–370. doi: 10.1507/endocrj.KR-80 17409575

[ref221] Young M. J. Clyne C. D. Cole T. J. Funder J. W. (2001). Cardiac steroidogenesis in the normal and failing heart. J. Clin. Endocrinol. Metab. 86, 5121–5126. doi: 10.1210/jcem.86.11.7925, PMID: 11701663

[ref222] Yu L. Cai H. Yue Q. Alli A. A. Wang D. Al-Khalili O.. (2013). WNK4 inhibition of ENaC is independent of Nedd4-2-mediated ENaC ubiquitination. Am. J. Physiol. Renal Physiol. 305, F31–F41. doi: 10.1152/ajprenal.00652.2012, PMID: 23594824 PMC3725674

[ref223] Zachar R. M. Skjødt K. Marcussen N. Walter S. Toft A. Nielsen M. R.. (2015). The epithelial sodium channel γ-subunit is processed proteolytically in human kidney. J. Am. Soc. Nephrol. 26, 95–106. doi: 10.1681/ASN.2013111173, PMID: 25060057 PMC4279735

[ref224] Zhang L. Chen L. Gao C. Chen E. Lightle A. R. Foulke L.. (2020). Loss of histone H3 K79 methyltransferase Dot1l facilitates kidney fibrosis by upregulating endothelin 1 through histone deacetylase 2. J. Am. Soc. Nephrol. 31, 337–349. doi: 10.1681/ASN.2019070739, PMID: 31843983 PMC7003297

[ref225] Zhang W. Xia X. Reisenauer M. R. Rieg T. Lang F. Kuhl D.. (2007). Aldosterone-induced Sgk1 relieves Dot1a-Af9-mediated transcriptional repression of epithelial Na+ channel alpha. J. Clin. Invest. 117, 773–783 (Comment in the same issue of *J. Clin. Invest*. 592–595, Selection by Faculty of 1000). doi: 10.1172/JCI29850, PMID: 17332896 PMC1804379

[ref226] Zhang W. Yu Z. Wu H. Chen L. Kong Q. Kone B. C. (2013). An Af9 cis-element directly targets Dot1a to mediate transcriptional repression of the alphaENaC gene. Am. J. Physiol. Renal Physiol. 304, F367–F375. doi: 10.1152/ajprenal.00537.2011, PMID: 23152297 PMC3566494

[ref227] Zhou J. Azizan E. A. B. Cabrera C. P. Fernandes-Rosa F. L. Boulkroun S. Argentesi G.. (2021). Somatic mutations of GNA11 and GNAQ in CTNNB1-mutant aldosterone-producing adenomas presenting in puberty, pregnancy or menopause. Nat. Genet. 53, 1360–1372. doi: 10.1038/s41588-021-00906-y 34385710 PMC9082578

[ref228] Zhou X. Chen K. Wang Y. Schuman M. Lei H. Sun Z. (2016). Antiaging gene klotho regulates adrenal CYP11B2 expression and aldosterone synthesis. J. Am. Soc. Nephrol. 27, 1765–1776. doi: 10.1681/ASN.2015010093, PMID: 26471128 PMC4884100

[ref229] Zhou R. Patel S. V. Snyder P. M. (2007). Nedd4-2 catalyzes ubiquitination and degradation of cell surface ENaC. J. Biol. Chem. 282, 20207–20212. doi: 10.1074/jbc.M611329200, PMID: 17502380

